# Influenza A, Influenza B, and SARS-CoV-2 Similarities and Differences – A Focus on Diagnosis

**DOI:** 10.3389/fmicb.2022.908525

**Published:** 2022-06-20

**Authors:** Andrei Havasi, Simona Visan, Calin Cainap, Simona Sorana Cainap, Alin Adrian Mihaila, Laura-Ancuta Pop

**Affiliations:** ^1^Department of Oncology, “Iuliu Hatieganu” University of Medicine and Pharmacy, Cluj-Napoca, Romania; ^2^Department of Medical Oncology, The Oncology Institute “Prof. Dr. Ion Chiricuta”, Cluj-Napoca, Romania; ^3^Department of Genetics, Genomics and Experimental Pathology, The Oncology Institute “Prof. Dr. Ion Chiricuta”, Cluj-Napoca, Romania; ^4^Pediatric Clinic No. 2, Department of Pediatric Cardiology, Emergency County Hospital for Children, Cluj-Napoca, Romania; ^5^Department of Mother and Child, “Iuliu Hatieganu” University of Medicine and Pharmacy, Cluj-Napoca, Romania; ^6^Faculty of Economics and Business Administration, Babes-Bolyai University, Cluj-Napoca, Romania; ^7^Research Center for Functional Genomics, Biomedicine and Translational Medicine, “Iuliu Hatieganu” University of Medicine and Pharmacy, Cluj-Napoca, Romania

**Keywords:** SARS-CoV-2, influenza, diagnosis, PCR diagnosis, COVID-19 influenza coinfection

## Abstract

In late December 2019, the first cases of viral pneumonia caused by an unidentified pathogen were reported in China. Two years later, SARS-CoV-2 was responsible for almost 450 million cases, claiming more than 6 million lives. The COVID-19 pandemic strained the limits of healthcare systems all across the world. Identifying viral RNA through real-time reverse transcription-polymerase chain reaction remains the gold standard in diagnosing SARS-CoV-2 infection. However, equipment cost, availability, and the need for trained personnel limited testing capacity. Through an unprecedented research effort, new diagnostic techniques such as rapid diagnostic testing, isothermal amplification techniques, and next-generation sequencing were developed, enabling accurate and accessible diagnosis. Influenza viruses are responsible for seasonal outbreaks infecting up to a quarter of the human population worldwide. Influenza and SARS-CoV-2 present with flu-like symptoms, making the differential diagnosis challenging solely on clinical presentation. Healthcare systems are likely to be faced with overlapping SARS-CoV-2 and Influenza outbreaks. This review aims to present the similarities and differences of both infections while focusing on the diagnosis. We discuss the clinical presentation of Influenza and SARS-CoV-2 and techniques available for diagnosis. Furthermore, we summarize available data regarding the multiplex diagnostic assay of both viral infections.

## Introduction

Influenza are negative-sense single-stranded RNA viruses, members of the Orthomyxoviridae family. Four influenza viruses are acknowledged within this family. Influenza A (IVA) and B (IVB) represent significant morbidity, and mortality causes in humans of all age groups and are responsible for local outbreaks and seasonal epidemics. Influenza infections are accountable for ∼500,000 deaths yearly and infect up to a quarter of the human population worldwide (Petrova and Russell, 2018). Influenza C viruses (IVC) can infect humans but usually cause mild disease in healthy adults; however, they may cause severe lower respiratory infections in children under 2 years old. In 2011, Influenza D viruses were identified as the newest members of the Orthomyxoviridae family, and the bovine species have been identified as a reservoir of infection. Several studies demonstrated that IDV could infect and spread among humans (Liu et al., 2020). Infection-associated symptoms may vary from mild upper respiratory tract involvement characterized by fever, rhinorrhea, cough, sore throat, muscle pain, headache, and fatigue to severe, potentially lethal pneumonia and non-respiratory complications involving the heart, central nervous system, and other organs leading to multiorgan failure or exacerbation of underlying conditions (Paules and Subbarao, 2017).

Human coronaviruses (HCoV) cause various respiratory conditions such as the common cold, bronchiolitis, and pneumonia (Pene et al., 2003). Additionally, HCoVs are associated with rapid disease progression due to increased proliferation rate via nucleotide substitution and recombination (Vijgen et al., 2005). Throughout the 21st century, HCoV’s have been identified in various locations worldwide and were correlated with outbreaks of deadly human pneumonia (Wu et al., 2020). The first CoV outbreak was reported in November 2002 in Foshan, China, causing severe acute respiratory syndrome (SARS-CoV) (Banerjee et al., 2019). In 2003, the outbreak spread into a global infection with a 10% mortality rate (Lee et al., 2003). In June 2012, the second pandemic caused by coronaviruses responsible for the Middle East Respiratory Syndrome (MERS-CoV) spread from Jeddah, Saudi Arabia, with a global mortality rate of 35% (de Groot et al., 2013). The third major pandemic caused by HCoV broke out in December 2019 in China’s Wuhan Province. It was caused by a new homologous strain of coronavirus (CoV-2) responsible for the severe acute respiratory syndrome (SARS-CoV-2), thus causing the Coronavirus disease (COVID-19) at a global level (Zhu et al., 2020). The pandemics caused by human coronaviruses are a continuous threat to human health and the world economy due to the high and unpredictable proliferation rate, leading to catastrophic consequences (Kirtipal et al., 2020).

The evolution of the current ongoing COVID-19 pandemic shows that SARS-CoV-2 is expected to continue to pose a critical healthcare concern in the years to come. Healthcare systems are likely to confront the overlapping of SARS-CoV-2 and influenza outbreaks.

The current review aims to underline the differences and similarities that these viruses share to provide a better understanding of pathogenesis, clinical manifestations, and treatment, focusing on the diagnosis of these infections.

## Virology

### Influenza

Influenza viruses are 80–120 nm enveloped filamentous or spherical, negative-sense single-stranded RNA viruses with a segmented genome containing eight RNA segments that encode several proteins, including the viral surface glycoproteins, which enable cellular entry – hemagglutinins (HA) and the release of new virions from the infected cells – neuraminidase (NA) (Cohen et al., 2013). The main three RNA segments encode the viral RNA-dependent RNA polymerase responsible for RNA synthesis and replication in the infected cells. RNA segment 5 encodes the viral nucleoprotein, which binds the RNA genome. The remaining segments encode several proteins: membrane protein (M1), non-structural protein (NS1), nuclear export protein (NEP), and several accessory viral proteins. Together these proteins regulate essential processes such as RNA segment rearrangement, viral entry and exit, virion genesis, and immune response evasion (Krammer et al., 2018). HA and NA viral proteins are located on the surface of the virus. They are the primary targets for the immune response. In the case of IVA, they allow classification in several antigenically diverse subtypes based on 16 distinct HA and nine different NA with the addition of two HA and NA identified in bats (Tong et al., 2013). However, just three HA subtypes of IVA (H1N1, H2N2, and H3N2) have caused pandemics in humans in the last century. Influenza B and C viruses do not display the same antigenic and genetic heterogeneity; IVB was recently classified into two distinct antigenic and genetic lineages, B/Victoria/2/1987 and B/Yamagata/16/1988 (Shaw et al., 2002).

Influenza viruses can spread from person to person through three mechanisms – aerosols, droplets, and contact transmission. Coughing and sneezing generate small aerosol particles that remain suspended in the air for various times ranging from minutes to hours, depending on changes in temperature and humidity. Currently, aerosol’s involvement in disease spreading is controversial, but the influenza genome has been identified in aerosols through polymerase chain reactions (PCR). Therefore it is safe to assume that aerosols from infected individuals may be inhaled and deposited in the upper or lower respiratory tract leading to disease transmission (Nikitin et al., 2014). Larger droplets usually fall within 3 m of the infected individual, infecting subjects situated in this range, and are generally deposited in the upper respiratory tract. Contact transmission may also occur. The virus can remain viable for various amounts of time depending on viral concentration, surface type, and environmental factors. Particles are then transferred to the mucous membranes of the upper respiratory tract leading to infection (Killingley and Nguyen-Van-Tam, 2013).

**FIGURE 1:**
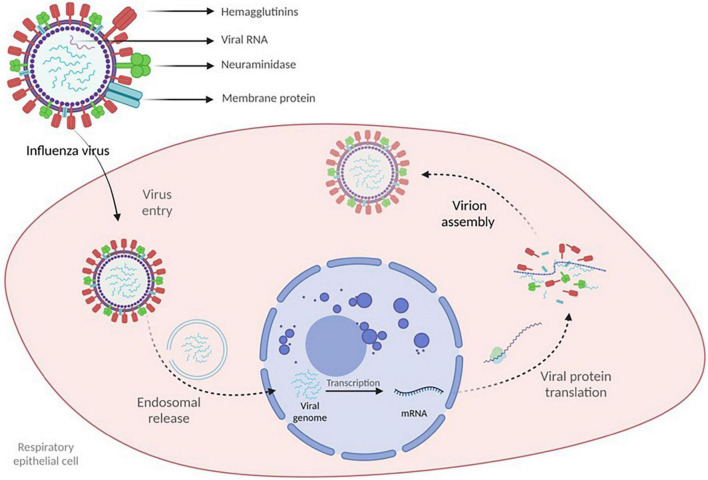
Influenza virus structure and viral genome synthesis.

Upon contact, the virus targets the epithelial cells of the respiratory tract and initiates viral replication. The virus binds with the epithelial cells via HA and is internalized in an endosome, leading to conformational changes in HA that eventually cause the release of viral genetic material into the cellular cytoplasm. The genetic material is then imported into the nucleus, where transcription and viral RNA replication are initiated, resulting in positive mRNA strands exported to the cytoplasm, where viral protein translation occurs with the generation of novel virions. NA enables the newly formed virions to leave the infected cell. Viral replication ends in cell death which, in conjunction with viral antigens, induces an inflammatory response aiming to eliminate the virus (Figure 1) (Krammer et al., 2018).

Influenza viruses possess two key traits that enable immune evasion: antigenic drift and antigenic shift. Antigenic drift refers to minor antigenic changes in viral HA and NA. Point mutations result in the accumulation of amino acid alterations in the antigenic sites of HA and NA surface glycoproteins. Antibodies generated by exposure to previous antigenic variants are unable to effectively neutralize the newly formed variant leading to the immunologic selection of a new predominant virus strain (Webster and Govorkova, 2014). At arbitrary time intervals, radical changes in the influenza virus antigenicity cause widespread disease and pandemics. These significant antigenic changes are called antigenic shifts and generate a viral strain toward which the population has little or no immunity. The segmented nature of the influenza genome allows the acquirement of HA or NA segments from various animal strains of Influenza through reassortment resulting in novel virus variants that encode completely different HA or NA, causing pandemic outbreaks responsible for substantial morbidity and mortality (Bouvier and Palese, 2008; Webster and Govorkova, 2014).

### SARS-CoV-2

The Coronaviridae family includes viruses with 80–220 nm length, a spherical or elliptical shape, enveloped, with peduncular prominences and rounded extremities arranged in a crown shape. The nucleocapsid has a helical symmetry with large spire loops and a monopartite genome organized in a linear mRNA, oriented in a positive sense and 27–32 kb in length (Figure 1). This family includes four genera: Alpha-, Beta-, Delta-, Gamma-coronavirus, and comprises more than 60 species with several viruses ranging from human to bovine, porcine, canine, feline, murine, leporid, and avian species of coronaviruses. However, a substantial number is found in bats. Members of this family of viruses infect various species, causing respiratory and digestive symptoms. Birds and bats play an essential role in spreading the coronaviruses. Some coronaviruses may cause zoonotic diseases responsible for largescale outbreaks (Perlman and Netland, 2009).

The diversity of the coronaviruses is the result of RNA polymerase inconsistency which is RNA-dependent polymerase, the increased frequency of homologous RNA recombination, and the size of the viral genome, the latter being the largest of all viral genomes with an RNA length of 27–32 kb. Due to the increasing number of coronaviruses, their adaptability to several host species, as well as the similarities between the viral genomes of a diverse range of species, it is worth considering that these viruses could be the cause of spreading zoonotic diseases such as SARS (Severe Acute Respiratory Syndrome) and MERS (Middle Eastern Respiratory Syndrome), which have recently evolved into human species (Woo et al., 2009; Rapuntean, 2019).

**FIGURE 2:**
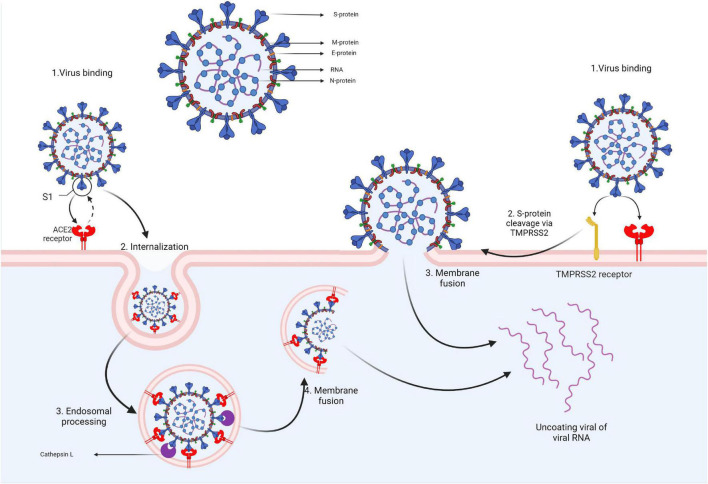
Coronavirus structure and cell entry mechanism.

The SARS-CoV-2 virion contains several structural proteins such as the spike (S) protein, membrane (M) protein, envelope (E) protein, and the nucleocapsid (N) protein that binds the genome RNA into a long helical ribonucleocapside. Upon contact with the target cell, viral entry is mediated via the interaction between the S protein and the angiotensin-converting enzyme 2 (ACE2) receptors. To enter the cell, the S protein must undergo a conformational transition that is enabled through the cleaving action of 2 proteases: cathepsin L and transmembrane protease, serine 2 (TMPRSS2). TMPRSS2 is expressed on the cell membrane while cathepsin L activation occurs in the endolysosome. Thus, based on the target-cell protease location, there are two cell entry pathways for SARS-CoV-2. If the target cell displays low TMPRSS2 expression, the virus-ACE2 complex is internalized through clathrin-mediated endocytosis into endolysosomes where S-protein cleavage occurs *via* cathepsin L. If the ACE2-virus complex is formed in the presence of TMPRSS2, cleavage occurs at the cell’s surface. S-protein cleavage leads to the fusion of cellular and viral membranes forming a fusion pore that enables viral RNA release into the target cell, thus enabling further RNA uncoating and replication (Figure 2) (Bai et al., 2021; Jackson et al., 2022).

## Clinical Presentation

### Influenza

Depending on both virus and host characteristics, the clinical presentation of Influenza ranges from asymptomatic infections to a fulminant illness. Typical Influenza usually begins with a sudden onset after an incubation period of 1–2 days. Systemic symptoms dominate the initial presentation and typically consist of fever, chills, headache, myalgia, malaise, and anorexia. Headache and myalgia involving extremities or back muscles are frequently the most troublesome symptoms, and their severity often correlates with the height of the fever. Ocular symptoms consisting of ocular muscle pain caused by lateral eye movement, tearing, lacrimation, and burning sensation are often present. Respiratory symptoms are also present at disease onset and consist of dry cough, pharyngeal pain, nasal discharge, and obstruction; however, they are often overshadowed by the systemic manifestations that distinguish Influenza from other upper respiratory tract infections. Children and older adults have different initial presentations. Children and infants have a higher fever, may present with febrile seizures, severe myalgia involving calf muscles, and often display gastrointestinal symptoms. Older adults present with high fever, fatigue, and confusion, sometimes without respiratory symptoms. Fever is the main finding at physical examination, and it can be as high as 4°C within 24 h of onset. Usually, fever is continuous and lasts for three days, but it may maintain high for up to 8 days. Upon fever remission, systemic symptoms diminish. Additional findings on physical examination are flushed facies, hot, moist skin, clear nasal discharge, hyperaemic nasal and throat mucosa, and small tender cervical lymphadenopathies (Cox and Subbarao, 1999; Rothberg et al., 2008; Paules and Subbarao, 2017; Bennett et al., 2019).

### SARS-CoV-2

SARS-CoV-2 infection exhibits a wide range of clinical presentations, often characterized by fever, dyspnea, lymphopenia, and lower respiratory tract infection (Nie et al., 2003). Although COVID-19 is considered mainly a viral pneumonia, the patients may also present gastrointestinal involvement such as diarrhea (30–40% of patients) caused by the active replication of SARS-CoV-2 in enterocytes (Leung et al., 2003), splenic atrophy, and gastrointestinal lymphadenopathy (To et al., 2004). In addition, infected individuals present slightly decreased platelet counts, prolonged coagulation profiles, and mildly elevated serum liver enzymes. The respiratory involvement caused by SARS-CoV-2 infection has been associated with diffuse alveolar lesions, epithelial cells proliferation, and increased macrophage infiltration. Multinucleate giant-cell infiltrates of macrophagic or epithelial origin have been associated with putative syncytium-like formation specific to most coronavirus infections. Symptoms like lymphopenia, hemophagocytosis, and white-pulp atrophy of the spleen observed in SARS-CoV-2 patients were reported to be similar to those identified in 1997 caused by the H5N1 influenza virus subtype outbreak (To et al., 2001). Although the disease may present with a wide array of clinical manifestations, some are more frequent than others. da Rosa Mesquita et al. performed a systematic review on the clinical manifestation of COVID-19 in the general population; it comprised data from 152 studies and over 41,000 patients. Six symptoms displayed higher prevalence: fever (58.66%), cough (54.52%), dyspnea (30.82%), malaise (29.75%), fatigue (28.16%), and sputum (25.33%). Neurological, dermatological manifestations, and anorexia were present in ∼20% of patients, while ∼10% had diarrhea, headache, and chest pain. Hemoptysis was the least reported symptom, only 1.65% of patients reporting hemoptysis (da Rosa Mesquita et al., 2021).

## Complications

### Influenza

The leading cause of influenza-associated mortality is the possible complications associated with infection. Two pulmonary complications are frequently associated with Influenza – primary Influenza viral pneumonia and secondary bacterial pneumonia. Primary, Influenza viral pneumonia starts with typical influenza symptoms followed in the first 24 h by rapid respiratory decompensation with severe dyspnoea, cyanosis, and hypoxemia. Critically diseased patients often develop adult respiratory distress syndrome and multiorgan dysfunction with increased mortality up to 80% of the cases (Rothberg et al., 2008; Bennett et al., 2019). Secondary, bacterial pneumonia evolves biphasic; symptoms resolve after the initial typical influenza presentation; however, fever recurs 4–14 days later and is usually associated with dyspnoea, productive cough, and consolidation on chest imaging. *Streptococcus pneumoniae, Staphylococcus aureus, Haemophilus influenzae*, other *Streptococcus* species, and other Gram-negative bacilli are the most commonly isolated pathogens from Influenza infection (Cox and Subbarao, 1999; Rothberg et al., 2008; Bennett et al., 2019). The incidence of bacterial coinfection of influenza varies across studies. One metanalysis and systematic review, including 3,215 patients across 27 studies, found high heterogeneity in bacterial coinfection rates ranging from 2 to 65%; however, in 64% of all patients, bacterial coinfection incidence ranged from 11 to 35%. *S. pneumoniae* and *S. aureus* were most frequently responsible, accounting for 35% respectively, 28% of all pathogens responsible for coinfection (Klein et al., 2016). Other pulmonary complications associated with Influenza are represented by bronchiolitis, exacerbation of asthma, and chronic bronchitis. One study found influenza accountable for 25% of all underlying chronic obstructive pulmonary disease exacerbations related to upper or lower inspiratory tract infections (Rohde et al., 2003). Besides pulmonary complications, the involvement of other organs and systems has been linked with flu. Myositis occurs more frequently in children than adults and is often associated with IVB infections. Most patients will have transiently elevated serum creatine phosphokinase, but some may present with important myoglobinuria and renal failure. More than 50% of hospitalized Influenza A patients presented elevated creatine phosphokinase in one study symptoms generally resolve after 4–6 weeks (Cox and Subbarao, 1999; Rothberg et al., 2008). Several neurologic manifestations that are associated with influenza infections include Reyes syndrome, Guillain-Barre syndrome, transverse myelitis, encephalomyelitis, aseptic meningitis, and encephalitis. Central nervous involvement is more frequent in children but causes a higher morbidity rate up to 30% in adult patients (Studahl, 2003; Tsai and Baker, 2013). Influenza can also be complicated with altered renal function. Renal influenza complications include acute kidney injury, acute glomerulonephritis, minimal change disease, and acute tubulointerstitial nephritis. Acute kidney injury may occur in 18–66% of intensive care unit patients leading to renal replacement therapy in up to 22% of cases. Liver injury has also been associated with influenza as up to 1/4 of patients may present with elevated AST and ALT (Sellers et al., 2017). Influenza more commonly causes exacerbation of underlying cardiac disorders; however, both pericarditis and myocarditis have been linked to Influenza (Bennett et al., 2019; Baral et al., 2020). Influenza-associated myocarditis has been reported in 0.4–13% of critically ill patients; disease severity ranges from asymptomatic to severe disease, with most patients presenting with cardiac symptoms rather than respiratory ones (Kodama, 2010; Sellers et al., 2017). Several reports also confirmed Influenza B-associated pericarditis (Horai et al., 2010; Spoto et al., 2019).

### SARS-CoV-2

Acute respiratory distress syndrome (ARDS) is one of the most frequent and potentially severe complications of SARS-CoV-2. A meta-analysis including 50,000 COVID-19 patients reported that 28.8% developed ARDS (Cao et al., 2020). ARDS causes diffuse alveolar damage responsible for hypoxemia, lung infiltrates, and fibrosis as the disease progresses. A prolonged inflammatory response and consequent epithelial damage are responsible for reducing the diffusing capacity of the lungs present in 30% of the patients one year after moderate SARS-CoV-2 infection. Lung fibrosis has also been associated with COVID-19. Although a more extended follow-up period is necessary, based on observational data from SARS, post-COVID lung fibrosis may be estimated at 2–6% for moderate to severe illness (Gibson et al., 2020; Bazdyrev et al., 2021). Other respiratory complications are secondary bacterial and fungal coinfections and sepsis which may be present in 8% of the cases (Rawson et al., 2020).

COVID-19 is also associated with several cardiovascular complications: myocardial injury and myocarditis, acute myocardial infarction, heart failure and cardiomyopathy, arrhythmias, and thromboembolic events. Myocarditis and myocardial injury may occur in 7–17% of hospitalized COVID-19 patients and up to 1/3 of those requiring intensive care. Heart failure may be present in 24% of patients; however, more data is necessary to distinguish if this is secondary to a SARS-CoV-2 induced cardiomyopathy or is caused by exacerbation of a previously undiagnosed heart condition. Arrhythmias are present in up to 44% of intensive care patients. These symptoms are frequently secondary to hypoxia, dyselectrolytemia, and inflammatory stress (Long B. et al., 2020). COVID-19 is also associated with an increased risk for thrombotic and thromboembolic events in up to 40% of patients. SARS-CoV-2 associated thrombosis is caused by several factors such as inflammatory response leading to endothelial dysfunction, sepsis, liver injury, intravascular coagulopathy, and bedridden stasis. These lead to venous thromboembolism, myocardial infarction, and disseminated intravascular coagulation (Bikdeli et al., 2020; Long B. et al., 2020).

**FIGURE 3:**
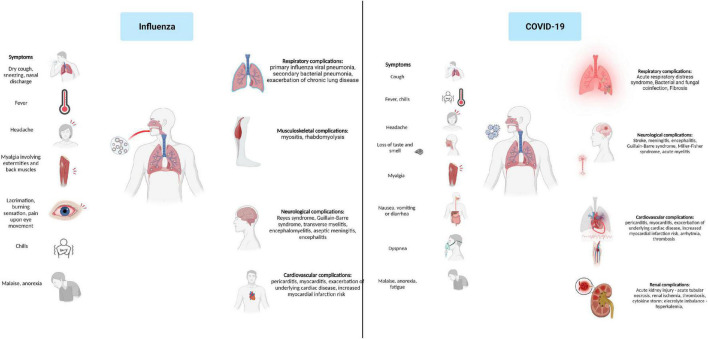
Influenza and COVID-19 clinical presentation and associated complications.

Neurological complications associated with COVID-19 have been reported in numerous studies. Hematogenous spread and retrograde neuronal dissemination are the primary mechanisms through which SARS-CoV-2 targets the neuronal cell leading to neurological manifestation that involve the central and peripheral nervous systems. Acute ischemic stroke has been reported in 2.1% of COVID-19 cases, followed by hemorrhagic stroke 0.4% and cerebral venous thrombosis 0.3%. Other severe neurologic complications associated with COVID-19 are meningitis, encephalitis, Guillain-Barre syndrome, Miller-Fisher syndrome, acute myelitis, and posterior reversible encephalitis syndrome. However, mild neurologic manifestations including gustatory, olfactory impairment, headache, myalgia, dizziness, and confusion were present in up to 82% of patients throughout the disease (Figure 3) (Favas et al., 2020; Harapan and Yoo, 2021).

Kidney involvement has been reported in COVID-19 patients. The incidence varies greatly across studies ranging from 0.5 to 46%, depending on the prevalence of preexisting chronic kidney disease and disease severity, with critically ill patients reporting a high incidence of kidney injury (Minami et al., 2020; Chan et al., 2021). A meta-analysis of 22 observational cohort studies encompassing over 17,000 COVID-19 patients found that 12.5% presented electrolyte imbalance, particularly hyperkalemia, 11% acute kidney injury, and 6.8% required renal replacement therapy (Kunutsor and Laukkanen, 2020). Acute kidney injury developed on average after nine days after admission. Age, diabetes, heart failure, and body mass index were associated with the risk and severity of acute kidney injury (Fanelli et al., 2020). Acute tubular necrosis is the most frequent histopathological finding associated whit SARS-CoV-2-related kidney involvement. Other possible mechanisms involved in COVID-19-associated kidney involvement are renal ischemia, cytokine storm, thrombotic complications, and rhabdomyolysis (Minami et al., 2020; Migliaccio et al., 2021).

## Diagnosis

### Influenza

In most cases, diagnosis is based on clinical presentation and epidemiological probability. Clinical diagnosis provides acceptable accuracy in healthy young and middle-aged adults presenting with acute influenza-like symptoms throughout seasonal epidemics. Several studies demonstrated an 80–90% accuracy of clinical diagnosis during influenza outbreaks (Boivin et al., 2000; Monto et al., 2000; Zambon et al., 2001). The diagnostic accuracy decreases in hospitalized, older patients and children due to atypical presentations and a higher probability of infection with other pathogens (Drinka et al., 2003; Ruest et al., 2003). The symptoms of Influenza are similar to the clinical presentation of other respiratory pathogens such as adenoviruses, coronaviruses, rhinoviruses, parainfluenza virus, respiratory syncytial virus, and specific laboratory diagnostic tests are needed to confirm influenza infection. Additionally, confirmation is essential for public health policies and epidemiologic surveillance (Newton et al., 2000; Petric et al., 2006).

There is a wide array of diagnostic tests available: rapid Influenza diagnostic tests, rapid molecular assay, immunofluorescence, direct or indirect fluorescent antibody staining, RT-PCR, rapid cell culture, and viral tissue cell culture [Information on Rapid Molecular Assays, RT-PCR, and other Molecular Assays for Diagnosis of Influenza Virus Infection CDC (Centers for Disease Control and Prevention [CDC], 2021b)].

In patients with influenza-like symptoms, samples should be ideally collected within 12–36 h from disease onset, allowing antiviral treatment initiation within the recommended 48-h timeline. Viral diagnostic testing has evolved considerably from the traditional viral cell cultures that rendered results in 10–14 days to rapid diagnostic tests that can confirm the diagnosis accurately in less than 30 min (Peaper and Landry, 2014; Bennett et al., 2019).

Several factors may influence results such as antigen variations, inadequate specimen – nasopharyngeal swabs, nasal aspirates, or lower respiratory samples are recommended while others may be suboptimal (sputum, throat, and nares swabs); improper timing – collecting too early (<12 h) or too late (>72 h) from symptom onset. Transport or storage may alter testing accuracy – improper storage – freezing, or prolonged storing can reduce viral titers or promote viral degradation. Excessive dilution in the transport media can alter rapid influenza test results. Other factors that may influence the accuracy of Influenza diagnostic tests are the different sensitivity and specificity of the chosen testing method test interpretation as rapid tests require reading at a specific time, and the direct fluorescence assays require subjective assessment [Rapid Influenza Diagnostic Tests | CDC (Peaper and Landry, 2014; Uyeki et al., 2019; Centers for Disease Control and Prevention [CDC], 2021c)].

### SARS-CoV-2

The management of the ongoing COVID-19 pandemic depends on providing accurate, accessible, time and cost-efficient testing. Currently, nucleic acid-based molecular diagnosis *via* real-time reverse transcription-polymerase chain reaction (RT-PCR) test is considered the golden standard for the early diagnosis of SARS-CoV-2 infection. However, this method requires adequate viral RNA concentrations in patient samples because of its high specificity. Research in qRT-PCR is focused on improving sensitivity, handling, time, and cost-efficiency (Benzigar et al., 2021). Sample source selection is a matter of debate as it may improve diagnostic efficiency. Lin et al. demonstrated higher detection rates in sputum samples than from throat swabs, 76.9% compared to 44.2% positive rates (*p* = 0.001). The SARS-CoV-2 ORF1b, E, and N genes were found to be specific to samples of human origin (Chu et al., 2020). Several laboratories developed qRT-PCR assays to detect the ORF1b and N gene and distributed these assays to improve SARS-CoV-2 detection worldwide (Sheridan, 2020). However, the fluctuations of mRNA levels in different tissues may lead to false-negative results (Feng et al., 2020). Suo et al. demonstrated that digital PCR might overcome the limitations of performing the assay in a low viral concentration setting. Nevertheless, this method has limitations regarding accessibility, cost, and lack of practical reliability (Suo et al., 2020). Alongside RT-PCR, antigen and antibody rapid testing is used to increase the current testing capacity. These rapid tests are readily available, cheap, can be used in a point of care setting, and render quick results but lack the sensitivity and specificity of nucleic acid amplification-based methods.

Sample quality is fundamental for the effective detection of SARS-CoV-2. The sample type, collection, and preprocessing play a vital part regardless of the technique. Nasopharyngeal and oropharyngeal swabs are frequently used for sample collection from the upper respiratory tract. However, these samples most often require a trained healthcare worker for specimen collection, increasing exposure risk. Saliva sampling is an alternative method because the patient can easily collect at home and provide higher detection rates compared to throat and nasal swabs (Fakheran et al., 2020). In addition, saliva sampling may facilitate the detection of both SARS-CoV-2 antigens and antibodies. One study found that saliva specimens contained more SARS-CoV-2 RNA copies than nasopharyngeal swabs, and there was less variation in nucleic acid levels throughout the clinical course of the disease. Furthermore, saliva specimens displayed higher sensitivity for SARS-CoV-2 detection in asymptomatic patients (Wyllie et al., 2020).

Sputum and bronchoalveolar lavage can also be used as samples for COVID-19 detection, with high detection rates. Blood and serum are most often used in antibody tests and tracking patients’ immune responses. Timing also plays a crucial role in sample source selection, with peak viral load from symptom onset in 0–7 days for nasopharyngeal swabs and 3–7 days for sputum and saliva (Jayamohan et al., 2021).

## Diagnostic Methods

### Rapid Influenza Diagnostic Tests

Rapid diagnostic tests are based on the immunologic identification of viral nucleoprotein antigens in respiratory secretions. Monoclonal antibodies target viral antigens using immunochromatographic or immunoassay techniques ensuing visual changes that allow the qualitative diagnosis of Influenza. RIDT can distinguish influenza type A and B, however, they cannot identify specific subtypes [Rapid Influenza Diagnostic Tests | CDC (Dzia̧bowska et al., 2018; Centers for Disease Control and Prevention [CDC], 2021c)]. The main advantage of RIDT is that they are easy to use and provide diagnostic in a point of care setting with a sensitivity ranging from 50 to 80% across studies and a specificity of 90% (McMullen et al., 2016; Dzia̧bowska et al., 2018). The number of viral antigens influences the sensitivity of RIDT. Up to 104–106 infectious particles are frequently required to ensure adequate sensitivity. RIDT sensitivity is highly dependent on viral shedding and is increased in children compared to adults (Casiano-Colón et al., 2003; Sakai-Tagawa et al., 2010). Another significant limitation to RIDT performance is antigenic variation. Several studies reported a considerable drop in sensitivity (40–60%) compared to circulating strains during the 2009 H1N1 influenza A virus outbreak (Centers for Disease Control and Prevention [CDC], 2021a). The variable performance of different manufacturers and the widespread use of RIDT has led to the reclassification of RIDT by the FDA as Class II *in vitro* devices and imposed specific performance criteria for all RIDT manufacturers. Currently, rapid influenza diagnostic tests must achieve minimum standards of 80% sensitivity for Influenza A and B compared to RT-PCR. Compared to viral cultures, a 90% sensitivity for influenza A and 80% for influenza B must be reached. Furthermore, the novel FDA classification requires manufacturers to test novel emerging strains within 30 days in the case of a public health emergency (Green and StGeorge, 2018).

### Rapid SARS-CoV-2 Diagnostic Tests

#### Antigen SARS-CoV-2 Testing

Rapid antigen diagnostic tests detect SARS-CoV-2 antigens in clinical samples collected from the respiratory tract of infected individuals. This testing method is based on the antibodies’ immune response against specific SARS-CoV-2 antigens found in the specimen. Testing kits utilize various immunological detection technologies such as lateral flow sandwich immunoassay, paramagnetic microbead-based immunoassay, and chromatographic digital immunoassay (Food and Drug Administration [FDA], 2022). The viral nucleocapsid is most often used as the target antigen due to its size, abundance in infected cells, and role as an immunodominant antigen in host response (Bai et al., 2021).

The optic lateral-flow immunochromatographic assay uses gold nanoparticles (AuNPs) and colorimetric labeling to provide a rapid platform for serologic testing in a point of care setting (Parolo et al., 2013). Specific SARS-CoV-2 antigens are conjugated to AuNPs, forming anti-human IgM and IgG conjugates that can detect human SARS-CoV-2 IgG and IgM from blood or saliva samples through colorimetric labeling. The assay renders results in 20 min with 100% sensitivity and 93.3% specificity compared to RT-PCR (Huang et al., 2020). Brümmer et al. analyzed data from 133 studies evaluating 61 different rapid antigen tests pooling a sensitivity and specificity of 71.2% (95% CI 68.2–74.0) and 98.9% (95% CI 98.6–99.1); however, when considering only manufacturer’s instructions conforming studies sensitivity increased to 76.3% (95% CI 73.1–79.2) (Brümmer et al., 2021). The World Health Organization (WHO) established a minimum performance requirement of ≥80% sensitivity and ≥97% specificity for antigen tests for SARS-CoV-2 diagnosis when RT-PCR testing is unavailable. Antigen testing is recommended for symptomatic individuals in the first 5–7 days after symptom onset. Their use in presymptomatic and asymptomatic cases is not recommended due to their low performance (World Health Organization [WHO], 2021).

#### Antibody SARS-CoV-2 Testing

Antibody synthesis against SARS-CoV-2 is the primary immune response to infection. Neutralizing antibodies are present in up to 50% of infected people by day 7 and in all infected people by day 14. Serological studies are an alternative to the diagnosis of SARS-CoV-2 by RT-PCR. Combining the RT-PCR technique with the serological testing significantly enhanced SARS-CoV-2 diagnosis. IgM levels increase in the first week after SARS-CoV-2 infection and peak after two weeks. IgG levels are detectable after one week and remain elevated for an extended time, sometimes over 48 days, and can serve as protection against reinfection (Hou et al., 2020). IgA responses occur 4 to 10 days after infection. Thus, serum IgA along with IgG and IgM, are considered COVID-19 diagnostic markers (Long Q. X. et al., 2020; Padoan et al., 2020). Antibody titers may decrease after seven days of illness. Recent studies have identified SARS-CoV-2 antibodies in saliva (Pisanic et al., 2020). Multiplex immunoassays for detecting SARS-CoV-2 antibodies have been used to determine the differences between antibody levels in saliva and serum. The simultaneous presence of antibodies in saliva and serum suggests a humoral-mediated immune response (Mahalingam et al., 2021).

Antibody testing differs concerning the types of antibodies measured: IgG, IgM, IgA, total immunoglobulins, or various combinations of the previously mentioned. Antibody assays generally target the SARS-CoV-2 nucleocapsid or spike protein. The most frequently used techniques for antibody detection are enzyme-linked immunosorbent assay (ELISA), chemiluminescence immunoassay (CLIA), and lateral flow immunoassays (Lai and Lam, 2021). Results are available in 13–45 min, depending on manufacturer and technique (Kevadiya et al., 2021). Although antibody testing provides good sensitivity and specificity, they have limited use in an acute infection setting. However, antibody testing can be used in conjuncture with antigen and nucleic acid testing to confirm the diagnosis when nucleic acid amplification and antigen tests have limited sensitivity. Antibody tests may also be used in seroprevalence studies to determine past SARS-CoV-2 exposures [Interim Guidelines for COVID-19 Antibody Testing | CDC (Safiabadi Tali et al., 2021; Centers for Disease Control and Prevention [CDC], 2022)].

### Real-Time Reverse Transcriptase PCR

#### Influenza Reverse Transcriptase-PCR Testing

Reverse transcriptase-PCR is regarded as the current gold standard for influenza diagnosis in most countries. RT-PCR is based on the simple principle of base pairing described by Watson and Crick. Firstly, viral RNA is reverse transcribed into complementary DNA, then the target gene is amplified using specific DNA primers and DNA polymerase. Finally, intercalating dyes or fluorescently labeled probes are added and detected by the equipment (Walper et al., 2018). Intercalating dyes bind to the amplified DNA and emit a fluorescent signal detected by the device. The main advantage of intercalating dyes is cost efficiency, however, this process has limited specificity and may lead to false-positive results (Courtney et al., 2021). Two fluorescent probes are most frequently used to diagnose Influenza: hydrolysis probes and molecular beacons. Both probes bind only specific DNA sequences allowing for increased accuracy, but they are more expensive than intercalating dyes. Hydrolysis probes are composed of a fluorophore and a quencher, which are detached *via* degradation during amplification. They have high specificity, reduced background fluorescence, and grant multiplex possibilities by using various fluorophores (Smith and Osborn, 2009). Molecular beacons consist of a fluorophore and a quencher, which are separated during amplification *via* displacement. Molecular beacons offer the same advantages as hydrolysis probes.

Additionally, they may allow allelic discrimination (Courtney et al., 2021). Time and cost are the main limitations associated with RT-PCR. In order to bypass these limitations, microwell PCR systems were designed to reduce reagent volumes from 20 to 5 μL (Ahrberg et al., 2019), and multiplex assays were developed to enable testing for multiple viruses or viral subtypes from a single sample (Dong et al., 2010).

Real-time reverse transcription-polymerase chain reaction (rRT-PCR) testing enables the detection and subtyping of influenza viral RNA from respiratory specimens *via* specific primers with high specificity and sensitivity, making it the current gold standard in influenza diagnosis (Gavin and Thomson, 2004). Several target genes are most commonly used for proper influenza virus identification *via* PCR, including but not limited to influenza type A matrix gene, hemagglutinin gene-specific for Influenza A subtypes A(H1N1)pdm09 virus, A(H3N2), former seasonal A(H1N1), the highly pathogenic avian influenza A (H5N1) virus, the avian influenza A(H7N9) virus, and other subtypes associated with zoonotic events (e.g., H9N2, H7Nx, H5Nx, and H10N8), type B influenza targeting the matrix, NP or NS genes of influenza B type-specific hemagglutinin genes. The accurate detection of Influenza *via* PCR is highly dependent on primer selection. To enable the amplification of the desired DNA region, a set of two primers are required; one primer is oriented in the 5′ → 3′ direction, the sense strand, and the other primer should complement the minus strand, which is oriented in the 3′ → 5′ direction, the antisense strand (Li and Brownley, 2010).

#### SARS-CoV-2 Reverse Transcriptase-PCR Testing

Currently, the diagnosis of SARS-CoV-2 is based on the detection of viral nucleic acids, antibodies, and proteins. Still, the real-time polymerase chain reaction (RT-PCR) remains the gold standard in diagnosing COVID-19 (Banerjee et al., 2019). Diagnosis based on nucleic acid detection is more sensitive and specific than currently available serological tests.

Nevertheless, RT-PCR testing for SARS-CoV-2 has some pitfalls that need improvement. A systematic review comprising 34 studies collecting data from 12,057 confirmed COVID-19 cases found a false-negative rate of 0.13 (95% CI 0.09–0.19); however, there was high heterogenicity between the included studies, with false-negative rates ranging from 0.018 to 0.58 (Arevalo-Rodriguez et al., 2020). Sampling timing is one of the key factors influencing false-negative results. Kucirka et al. found a false-negative rate one day before symptom onset of 67% (CI, 27–94%); this decreased to 20% (CI, 12–30%) 3 days after symptom manifestation, then started to rise again (Kucirka et al., 2020). Sample viral load also influences false-negative rates, with lower respiratory tract samples featuring higher loads than other sample sources (Abbasi-Oshaghi et al., 2020). Viral RNA denaturation or degradation may also occur due to improper sample manipulation or storage. False-negative rates can be lowered by considering clinical presentation and associating antibody testing (Teymouri et al., 2021). Additionally, to limit false-negative results, particularly in the setting of low viral loads, current guidelines recommend the simultaneous detection of at least two target SARS-CoV-2 genes (World Health Organization [WHO], 2020; Tombuloglu et al., 2022). Virus mutation rates also influence false-negative rates. Data from sequencing studies showed that SARS-CoV-2 has a moderate mutation rate of ∼two nucleotides/month. However, despite the low mutation rate, over 12,000 mutations in the SARS-CoV-2 genome have been identified (Callaway, 2020). Mutations in the primer binding site for nucleic acid amplification may hinder assay sensitivity. Several reports found that mutations in the E or N SARS-CoV-2 gene interfered with virus detection leading to false-negative results (Artesi et al., 2020; Ziegler et al., 2020; Hasan et al., 2021). To overcome this limitation, the use of primers directed at multiple target genes is recommended by current guidelines (Food and Drug Administration [FDA], 2021). Other ways to surpass the impact of new mutation reside in better primer design. Dong et al. developed a primer design strategy based on nucleic acid sequence and the three-dimensional structure of the encoded protein. Using this principle, they designed primer pairs that targeted the nucleocapsid (N) gene. Their design rendered similar sensitivity and specificity to the US and Chinese CDC-validated primers, but more importantly, performance was not influenced by frequently occurring mutations (Dong et al., 2021).

False-positive results can also decrease RT-PCR diagnostic accuracy. Misleading results may occur due to cross-contamination, inactive viral RNA remnants, detection of other coronaviruses, or technical reasons relating to primers, probes, or procedures (Mouliou and Gourgoulianis, 2021). After patient discharge, a positive RT-PCR assay is challenging in differentiating between a false-positive assay due to the shedding of inactive SARS-CoV-2 remnants, reinfection, or reactivation. Multiple studies underlined the possibility of SARS-Cov-2 reinfection or reactivation however, there is little data on accurately distinguishing the two scenarios. Tang et al. performed a systematic review that included data from studies analyzing more than 113,715 patients to solve this conundrum. They observed that if the interval between discharge and positivity is ≤28 days, reinfection or relapse is more plausible- with reactivation generally occurring ≤15 days; if the time interval is 2 months, it is more likely to be reinfection, and if the time from discharge is longer than 3 months then reinfection is very likely to be the cause. Nevertheless, the most reliable way to distinguish reinfection from reactivation is to perform whole-genome sequencing to assess if the newly detected SARS-CoV-2 is a different strain (Siqueira et al., 2021; Tang et al., 2021). It has been shown that viral shedding can continue long after the immune system neutralizes the virus and is no longer infectious. Shedding duration varies across reports; one meta-analysis investigating data from 79 studies reported a mean shedding duration of 17 days with a maximum shedding in the upper respiratory tract of 83 days. No study managed to find a live virus beyond day 9 of illness despite high viral loads (Cevik et al., 2021). However, RT-PCR may detect viral RNA remnants rendering false-positive results. Thus, a differential diagnosis between reinfection and viral shedding is needed. Several approaches are available to distinguish the two: genome sequencing may be employed, a distinct SARS-CoV-2 variant would provide a definitive diagnosis of reinfection, while the identification of the same variant requires additional testing if clinical suspicion of reinfection is high. Viral load quantification – one study found that viral loads below 6.6310RNA copies/mL are associated with a less than 5% probability of isolating infectious virus (van Kampen et al., 2021). Viral cultures can also provide information in this regard – if viral cultures are negative, then viral shedding is more likely while identifying viable viruses argues for reinfection or reactivation. Clinical features may also aid in the differential diagnosis: the elapsed time from the first RT-PCR confirmation – longer time intervals being associated with a higher probability of reinfection; the presence of symptoms is also likely to suggest reinfection most patients with prolonged viral shedding are asymptomatic (European Centre for Disease Prevention and Control [ECDC], 2020; Tuan et al., 2021).

PCR virus detection is performed based on a known and relatively efficient protocol with high accuracy. However, increased mutation rates are responsible for major viral nucleic acid structure changes, thus lowering the PCR-based test’s diagnostic power. In order to surpass this limitation, the use of specific primers and probes is recommended. A variety of RT-PCR assays using various primers and probes have been developed. A report by Anantharajah et al. compared the clinical performance of the primer/probe sets recommended by the WHO. A significant difference was found in SARS-CoV-2 detection between different primer/probes (Anantharajah et al., 2021). Mollaei et al. compared five primer sets’ accuracy in SARS-CoV-2 detection *via* RT-PCR. Primers targeting ORF1ab, nucleocapsid (N), and RNA-dependent RNA polymerase (RdRp) genes had higher sensitivity, specificity, and positive predictive value compared to those aimed at the spike protein (S) and envelope (E) genes (Mollaei et al., 2020). Another study performed by the University of Washington Clinical Virology Lab analyzed seven different primer/probe sets. They found the primers targeting the E gene proposed by Corman et al. (2020) to be the most sensitive (Nalla et al., 2020).

Isothermic amplification techniques are an alternative to cyclic nucleic acid amplification. Simplified RT-PCR testing is available for different regions of the SARS-CoV-2 genome. The RdRp, helicase (Hel), and nucleocapsid genes are commonly targeted *via* RT-PCR, with RdRp/Hel diagnostic tests rendering higher viral RNA detection sensitivity. High-output platforms such as Cobas 6800, alongside proper sample manipulation, ensure quick and accurate COVID-19 diagnosis (Eigner et al., 2019; Zhang W. et al., 2020).

RT-PCR testing remains the gold standard for SARS-CoV-2 diagnosis. Despite high accuracy, the assay is limited by equipment and personnel requirements and prolonged procedure time. Depending on the protocol, the process is completed in 4–8 h. To overcome this limitation, automated or semi-automated high throughput platforms have been developed to analyze a large number of samples while also limiting reagents costs (Falzone et al., 2021). A different approach to overcoming RT-PCR testing limitations was the development of rapid diagnostic tests based on the RT-PCR principles. These assays provide an accurate diagnosis with minimal hands-on time in less than 1 h, thus enabling their use in a point-of-care setting. The Xpert Xpress SARS-CoV-2 assay on the Cepheid GeneXpert platform renders results in <45 min with minimal hands-on time while providing an accurate and sensitive diagnosis. One systematic review encompassing data from 14 studies and 1,647 samples found a sensitivity and specificity of 0.97 (0.96–0.98) and 0.97 (0.96–0.98) and an AUC of 0.9926 for the Xpert Xpress SARS-CoV-2 (Cao et al., 2022). Loeffelholz et al. performed a multicenter evaluation of Xpert Xpress SARS-CoV-2 accuracy compared to standard of care RT-PCR testing using 483 clinical samples. The positive agreement rate was 99.5%, while the negative agreement was 95.8%. A third nucleic acid amplification test was used to analyze the discordant results, all in favor of the Xpert assay (Loeffelholz et al., 2020). Similarly, Roche’s cobas Liat SARS-CoV-2 assay provides accurate diagnosis in 20 min with high accuracy when compared to standard of care RT-PCR assays. A multicentric analysis of cobas Liat SARS-CoV-2 performance using samples from 444 patients demonstrated a 100% positive agreement rate and 97.4% negative agreement rate (Hansen et al., 2021).

However, despite promising high accuracy and significantly lower assay time, these findings need further validation in a real-world clinical practice setting. Most of these results are primarily based on remnant laboratory samples, and there is little data on their accuracy in relation to sample collection timing or patient symptom status. Prospective and comparative analysis in clinically relevant settings is needed in order to validate these assays properly. Until then, they can be considered when there is a need to make a quick decision about the patient or in situations where RT-PCR cannot be performed in a timely manner.

#### Multiplex Reverse Transcriptase-PCR Diagnosis of Influenza and SARS-CoV-2

Patients infected with SARS-CoV-2 can display various clinical presentations ranging from asymptomatic to severe acute respiratory distress syndrome. However, most patients present with influenza-like symptoms such as fever (99%), chills, dry cough (59%), fatigue (70%), lethargy, arthralgia, myalgia (35%), headache, dyspnea (31%), anorexia (40%), thus making a differential diagnosis, solely based on clinical presentation, challenging (Liu and Pan, 2020; Song et al., 2020). Infection confirmation is necessary *via* real-time reverse transcription-polymerase chain reaction to establish an accurate diagnosis and ensure proper therapeutic management (Valencia, 2020).

Shu et al. developed the Centers for Disease Control and Prevention (CDC) Influenza SARS-CoV-2 Multiplex Assay to address this issue, enabling the simultaneous detection of influenza A, B, and SARS-CoV-2 from upper and lower respiratory samples. The primers used for influenza detection were identical to those used in the singleplex assays of the US Food and Drug Administration (FDA)–approved CDC Human Influenza Virus Real-Time RT-PCR Detection and Characterization Panel. The assay rendered high sensitivity and specificity; it detected Influenza A virus at 102.0, influenza B virus at 102.2, and SARS-CoV-2 at 100.3 50% tissue culture or egg infectious dose, or as few as 5 RNA copies/reaction. The assay was evaluated with viral RNA from 13 influenza A and two influenza B isolate to assess primer specificity. No cross-reactivity could be observed. Additionally, assay performance was evaluated using nucleic acids from 104 clinical samples. In all instances, the results agreed with the expected value for each specimen (Shu et al., 2021). Minjun et al. also evaluated a one-step quadruplex rRT-PCR assay to detect and differentiate SARS-CoV-2 ORF1ab and N genes, influenza type A and B. Primers targeting the matrix protein gene and the neuraminidase gene were designed to enable the detection of influenza A and B viral RNA (Table 2). The assay’s performance was assessed using 312 clinical samples consisting of 110 nasopharyngeal swabs, 186 oropharyngeal swabs, and 16 sputum samples. Results were compared to singleplex rRT-PCR assays. Only four tested clinical samples displayed inconsistent results with the singleplex rRT-PCR assays. However, the quadruplex detected Influenza A and B RNA with 100% sensitivity and specificity, and all the results agreed with the expected outcome (Ni et al., 2021).

**TABLE 1:** Data on current RT-PCR multiplex detection of SARS-CoV-2, Influenza A, B, and Respiratory syncytial virus.

References	Assay	Platform	Target virus	Limit of detection	Sensitivity (%)	Specificity	Clinical sample size
Chung et al. (2021a)	BD MAX dual multiplex real-time RT-PCR panel	BD MAX	SC2	50 copies/PCR	100%	100%	205
			IFV A	100–200 copies/PCR	N/A	N/A	
			IFV B	100 copies/PCR	N/A	N/A	
			RSV	100 copies/PCR	N/A	N/A	

Kim et al. (2022)	PowerChek SARS-CoV-2, Influenza A & B Multiplex Real-time PCR Kit	PowerChek	SC2	0.16 copies/μL	97.5%	100%	147
			IFV A	0.14 copies/μL	100%	100%	
			IFV B	0.034 copies/μL	100%	100%	

Nörz et al. (2021)	SC2/InflA/InflB-UCT	Cobas6800	SC2	94.9 copies/mL^–1^	98.1%	N/A	164
			IFV A	14.57 copies/mL	97.7%	N/A	
			IFV B	422.3 copies/mL^–1^	100%	N/A	

Pabbaraju et al. (2021)	SC2/Flu (SARS-CoV-2/influenza A and B) assay	easyMAG, MagMAX Express 96, Hamilton STARlet	SC2	3 copies/PCR	100%	99.83%	128
			IFV A	2 copies/PCR	100%	100%	
			IFV B	2 copies/PCR	100%	98.86%	

Chung et al. (2021b)	SARS-CoV-2, influenza A/B, RSV in multiplex RT-PCR	LabTurbo AIO 48	SC2	9.4 copies/PCR	N/A	N/A	652
			IFV A	24 copies/PCR	N/A	N/A	
			IFV B	24 copies/PCR	N/A	N/A	
			RSV	24 copies/PCR	N/A	N/A	

Kim et al. (2021)	PowerChek SARS-CoV-2, Influenza A&B, RSV Multiplex Real-time PCR Kit	PowerChek	SC2	0.36 copies/μL	100%	100%	175
			IFV A	1.24 copies/μL	100%	100%	
			IFV B	0.09 copies/μL	100%	100%	
			RSV	0.63 copies/μL	93.1%	100%	

Kim et al. (2021)	Allplex™ SARS-CoV-2/FluA/FluB/RSV Assay	Allplex	SC2 -N gene	1,649.6 copies/mL	95.9%	100.0%	403
			SC2 - RdRp gene	283.5 copies/mL			
			SC2 -S gene	361.0 copies/mL			
			IFV A	4,917.3 copies/mL	100%	99.7%	
			IFV B	248.9 copies/mL	100%	100%	
			RSV	282.48 copies/mL	94.0%	100%	
	STANDARD M Flu/SARS-CoV-2 Real-time Detection Kit	STANDARD M	SC2 – E gene	1,176.4 copies/mL	95.9%	100.0%	
			SC2 - ORF1ab gene	259.7 copies/mL			
			IFV A	11,205 copies/mL	100%	99.7%	
			IFV B	578.0 copies/mL	100%	100%	
	PowerChek SARS-CoV-2, Influenza A&B Multiplex Real-time PCR Kit	PowerChek	SC2 – E gene	212.1 copies/mL	92.8%	100%	
			SC2 - ORF1ab gene	402.3 copies/mL			
			IFV A	5,661.8 copies/mL	100%	100%	
			IFV B	88.8 copies/mL	100%	100%	

Paradis et al. (2021)	MAX SARS-CoV-2/Flu	BD MAX	SC2	N/A	96.2%	100%	
			IFV A	N/A	100%	98.9%	235
			IFV B	N/A	98.3%	100%	

Trick et al. (2021)	Rapid multiplexed screening of SARS-CoV-2/Flu	Magnetofluidic cartridge platform for automated PCR	SC2	2 copies/μL	98.1%	95.2%	130
			IFV A	2 copies/μL	87.5%	100%	
			IFV B	24 copies/μL	100%	98.2%	

Mboumba Bouassa et al. (2022)	AMPLIQUICK^®^ Respiratory Triplex	AMPLIQUICK	SC2	N/A	97.6%	100%	442
			IFV A	N/A	97.9%	100%	
			IFV B	N/A	89.5%	100%	
			RSV	N/A	100%	100%	

Shu et al. (2021)	Influenza SARS-CoV-2 Multiplex Assay	Thermo Fisher	SC2	5 copies/PCR	100%	100%	104
			IFV A	5 copies/PCR	100%	100%	
			IFV B	5 copies/PCR	100%	100%	

Cheng et al. (2022)	Abbott Alinity m Resp-4-Plex assay	Alinity m	SC2	≤25 copies per mL	N/A	N/A	72
			IFV A	47 copies/mL	N/A	N/A	
			IFV B	36 copies/mL	N/A	N/A	
			RSV	39.8 copies/mL	N/A	N/A	

Mancini et al. (2021)	Multiplex rtRT-PCR for SC2 and seasonal flu	Roche BioRad Stratagene Rotor Gene Q Applied Biosystems Bioline	SC2	N/A	98.8%	100%	1000
			IFV A	N/A	100%	100%	
			IFV B	N/A	100%	100%	

Zhen et al. (2022)	Alinity m Resp-4-Plex Assay	Alinity m	SC2	26 copies/mL	95%	100%	100
			IFV A	36 copies/mL	100%	100%	
			IFV B	22 copies/mL	95%	100%	
			RSV	22 copies/mL	100%	100%	
	Xpert Xpress SARS-CoV-2, flu A/B, and RSV	GeneXpert Xpress	SC2	83 copies/mL	100%	100%	
			IFV A	32 copies/mL	100%	100%	
			IFV B	38 copies/mL	95%	100%	
			RSV	326 copies/mL	95%	100%	
	Cobas Liat SARS-CoV-2 and flu A/B	Cobas Liat	SC2	58 copies/mL	95%	100%	
			IFV A	77 copies/mL	100%	100%	
			IFV B	122 copies/mL	95%	100%	

Wolters et al. (2021)	Xpert Xpress SARS-CoV-2/Flu/RSV	GeneXpert Xpress	SC2	N/A	97.2%	100%	295
			IFV A	N/A	95.3%	100%	
			IFV B	N/A	95.6%	100%	
			RSV	N/A	96.1%	96.8%	

*SC2, SARS-CoV-2; IFV A, Influenza A; IFV B, Influenza B; RSV, Respiratory syncytial virus.*

**TABLE 2:** SARS-CoV-2 diagnostic assays advantages and limitations.

Assay technique	Advantages	Limitations
Rapid antigen test	Assay time – min; Suitable for point of care setting use; Easy to use, visual readout; Cost-efficient;	Lower specificity and sensitivity compared to RT-PCR Low performance in asymptomatic and presymptomatic patients
Rapid antibody test	Assay time – min; Easy to use;	Not suitable for diagnosis; Can be used retrospectively; Cross-reactivity;
RT-PCR	High sensitivity & sensitivity; Capable of high throughput through designated platforms; Current gold standard; Multiplexing	Requires equipment, trained personnel, and reagents cost; Hard to implement in a point of care setting; Assay time – hours;
LAMP	High sensitivity & specificity; Suitable for point of care use; Assay time ∼1 h; Cost-efficient; Multiplexing;	Complex primer design; Higher false-positive rate compared to RT-PCR;
CRISPR	High sensitivity & specificity; Assay time < 1 h; Cost-efficient; Multiplexing;	Laks extensive validation for SARS-CoV-2;
NGS	Viral strain identification and characterization; Phylogenetic tracing; Information regarding treatment resistance, vaccine efficiency;	Requires complex equipment, highly trained personnel; Expensive; Long assay time;

One study adapted a laboratory-developed multiplex RT-PCR assay for simultaneous detection of SARS-CoV-2, influenza A and influenza B on a fully automated high-throughput system, Cobas 6800 system, a fully automated sample-to-result high-throughput platform, requiring minimal hands-on time and able to perform up to 384-tests in an 8-h shift. Assay performance was assessed *via* serial dilution of quantified reference material and cell culture stocks in transport medium and evaluation of predetermined clinical samples. Custom-made primers were used for the detection of influenza A and B RNA. The assay enabled the detection of Influenza nucleic acids with 97.7% sensitivity for influenza A and 100% sensitivity for influenza B. In cross-reactivity experiments, there were no false-positive results. The assay was able to detect Influenza A H1N1 pdm09 (A/Michigan/45/2015), Influenza A H7N9 (A/Anhui/1/2013), Influenza A H5N8 (A/DE-SH/Reiherente/AR8444/2016), Influenza B Yamagata (B/Phuket/3073/2013), and Influenza B Victoria (B/Colorado/06/2017) strains (Nörz et al., 2021). A similar study also evaluated a high-throughput platform multiplex rRT-PCR for the simultaneous detection of SARS-CoV-2, influenza A, B, and respiratory syncytial virus. The BD MAX platform multiplex was validated using 205 pretested clinical samples. Results were consistent with the gold standard approved singleplex assay and accurately detected influenza A and B infections. Furthermore, the BD MAX platform shortened turnaround time to 2.5 h by running 24 samples per batch and 192–216 samples in 11 h (Chung et al., 2021a). Similarly, Pabbaraju et al. validated a multiplex rRT-PCR assay for the simultaneous detection of SARS-CoV-2, influenza A and influenza B. Detection of Influenza A and B was performed using rtRT-PCR assays targeting the matrix (M) and non-structural protein 1 (NS1) genes of influenza A and B, respectively. The assays have been developed by the CDC and approved by the FDA to diagnose seasonal Influenza A, B, H1, H3, and avian H5 serotypes. Multiplex sensitivity and specificity were evaluated in comparison to gold standard singleplex assay using 128 clinical samples and rendered a sensitivity and specificity of 100% (95% CI: 90.8–100%), (95%CI: 94.03–99.97%) for influenza A; for influenza B 100% sensitivity (95%CI: 91.96–100%) and 98.9% (95%CI: 93.8–99.97%) specificity; while for SARS-Cov-2 the multiplex assay assured a 100% (95%CI: 93.3–100%) sensitivity and 99.83% (95%CI: 99.1–100%) specificity(Pabbaraju et al., 2021).

In order to analyze currently available data on multiplex RT-PCR detection of Influenza and SARS-CoV-2, we performed a PubMed database search using [(Influenza) AND (SARS-CoV-2)] AND (Multiplex PCR). We selected articles in English. Of the initial 64 results, we selected 15 studies. We summarized the findings regarding assay limit of detection (LoD), sensitivity, specificity, target virus, and clinical validation sample data in Table 1.

## Isothermal Amplification Techniques

Isothermal amplification techniques are performed at a single reaction temperature under simple conditions, and therefore, they are more cost-efficient, quicker, and more energy-efficient than the classic thermal cycling procedure. Several isothermal amplification techniques have been applied in diagnosing Influenza and SARS-CoV-2, allowing an accurate diagnosis in a point of care setting and resource-limited settings.

### Loop-Mediated Isothermal Amplification – Influenza

Loop-Mediated Isothermal Amplification (LAMP) uses inner and outer loop primer pairs to hybridize and amplify a specific cDNA sequence. The primer pairs amplify the target sequence through self-hybridization within the newly amplified strands resulting in dumbbell-shaped amplicons, which provide loop primer binding sites. Reverse transcriptase and DNA polymerase are contained within the reaction mixture for influenza viruses. Amplified DNA is quantified *via* fluorescent signals emitting intercalating dyes. LAMP is cost-effective; using isothermal conditions only requires a heating block for amplification, offers high sensitivity and specificity >95%, and can be used in multiplex panels. However, the main limitation associated with LAMP is primer design, a time-consuming, complex process requiring high expertise (Kubo et al., 2010; Abe et al., 2011; Mahony et al., 2013).

### Loop-Mediated Isothermal Amplification – SARS-CoV-2

Loop-mediated isothermal amplification, in association with reverse transcription (RT-LAMP), is the most frequently used isothermal amplification technique for SARS-CoV-2 detection. RT-LAMP targets specific regions of the viral genome by using 4–6 primers and DNA polymerase, thus enabling the detection of ORF1ab, S, E, and N genes of the SARS-CoV-2. RT-LAMP amplifies target DNA isothermally, and results are measured photometrically using fluorescent or colorimetric dyes. Turnaround time is ∼1 h, and the limited equipment requirements alongside higher cost-efficiency make it a good candidate for accurate point of care testing (Mardian et al., 2021). Furthermore, RT-LAMP may also be performed at home. The Accessible LAMP-Enabled Rapid Test (ALERT) for SARS-CoV-2 detection is an affordable (<5 $), five-stage assay that enables viral nucleic acid detection with high sensitivity (LoD as low as 0.1–2 copies/μL) and specificity (97%). In addition, results are available in 60 min, and the flexible nature of ALERT allows the co-detection of other respiratory viruses while the easy workflow enables wide use (Bektaş et al., 2021). A meta-analysis of 33 studies encompassing 9,360-suspected SARS-CoV-2 cases compared the screening value of RT-PCR and RT-LAMP. Overall pooled sensitivity was 96% for RT-PCR and 92% for RT-LAMP. RT-PCR specificity was 100%, while RT-LAMP had a 99% specificity for the diagnosis of SARS-CoV-2. False-negative and false-positive rates for RT-LAMP were 12 and 1%, respectively (Pu et al., 2022).

### Loop-Mediated Isothermal Amplification Multiplex for SARS-CoV-2 and Influenza

Lee et al. developed an RT-LAMP-based multiplex assay aiming to detect SARS-CoV-2, Influenza A, and B in a point of care setting. The assay detected viral RNA reliably with a LoD of 50 copies/μL. Saliva samples analyzed through qRT-PCR confirmed COVID-19 diagnosis in the patients that were used to validate the assay clinically. The multiplex panel was in 100% concordance with PCR testing (Lee et al., 2021). Similarly, Zhang et al. also described an RT-LAMP-based method for the multiplex detection of SARS-CoV-2 and Influenza. The assay was not only able to identify the presence of each viral RNA but managed to distinguish coinfection with multiple viruses (Zhang and Tanner, 2021).

### CRISPR – Influenza

Recombinase polymerase amplification (RPA) and CRISPR-based diagnostics clustered regularly interspaced short palindromic repeats (CRISPR)-Cas (CRISPR-associated proteins) systems have been recently developed to detect influenza infections. The specific high-sensitivity enzymatic reporter unlocking (SHERLOCK) consists of isothermal RPA followed by transcription and detection of the target RNA using the Cas13 nuclease and was shown to detect the Influenza A virus with high accuracy (Freije et al., 2019). An additional CRISPR-Cas13 technique labeled Combinational Arrayed Reactions for Multiplexed Evaluation of Nucleic Acids-Cas13 (CARMEN-Cas13) was able to detect 169 human viruses simultaneously with attomolar sensitivity. Furthermore, CARMEN-Cas13 was able to identify the Influenza A virus and enable subtyping for H1–H16 and N1–N9. It also detected several drug resistance mutations in HIV and swiftly incorporated CRISP RNA to detect the causative agent of the COVID-19 pandemic; due to its highly multiplex characteristics, including high specificity and accuracy, this technique leads to a 300 fold decrease in reagent cost (Ackerman et al., 2020).

### CRISPR – SARS-CoV-2

CRISPR- based detection methods have been developed for the diagnosis of SARS-CoV-2. Chen et al. combined the activation of Cas12a single-stranded deoxyribonuclease with isothermal amplification to create the DNA endonuclease-targeted CRISPR trans reporter (DETECTR) technique. DETECTR quickly and accurately detected human papillomavirus in human samples (Chen et al., 2018). Mammoth Biosciences adapted this technique to enable accurate and specific SARS-CoV-2 detection in response to the ongoing pandemic. DETECTR coupled with RT-LAMP provided high-precision results in a low-resource setting within 30 min (Ali et al., 2020). Broughton et al. compared the DETECTR RT-LAMP assay with the CDC qRT-PCR assay for SARS-CoV-2 detection; results were validated using samples from 36 COVID-19 patients and 42 samples with other respiratory viruses. DETECTR RT-LAMP rendered results in 45 min vs. 4 h for RT-PCR with a detection limit of 10 copies/μl compared to 1 copies/μl for PCR, a 95% predictive agreement and 100% negative predictive agreement (Broughton et al., 2020). The specific high-sensitivity enzymatic reporter unlocking (SHERLOCK) technique has also been adapted for SARS-CoV-2 detection (Zhang F. et al., 2020). SHERLOCK-based SARS-CoV-2 diagnosis was clinically validated using 534 nasopharyngeal and throat samples. Lateral flow and fluorescence readout SHERLOCK were compared to qRT–PCR. LoD was 42 copies/reaction. The assay was 100% specific and 96% sensitive with the fluorescence readout, while the lateral-flow readout had 86% sensitivity (Patchsung et al., 2020). Compared to the DETECTR technique, SHERLOCK has higher detection sensitivity of 10–100 copies/μl compared to the 70–300 copies/μl of the input sample. Additionally, unlike DETECTR, SHERLOCK can regulate the expression profile of genes without modifying the genome. Therefore, the flexibility, robustness and sensitivity provide an advantage for SHERLOCK (Gupta et al., 2021). A third assay developed by Azhar et al. identifies viral RNA using the Cas9 nuclease. FNCAS9 Editor Linked Uniform Detection Assay (FELUDA) combined with lateral flow readout provided accurate SARS-CoV-2 diagnosis with a 100% sensitivity and 96.7% specificity compared to qRT-PCR on 473 clinical samples (Azhar et al., 2021).

### CRISPR Multiplex for SARS-CoV-2 and Influenza

Furthermore, Welch et al. developed a multiplexed microfluidic CRISPR-based platform, mCARMEN, which enabled the diagnosis of 21 respiratory viruses, including SARS-CoV-2 and Influenza. mCARMEN was further enhanced to identify six SARS-CoV-2 variants, including Delta and Omicron. mCARMEN variant identification panel provided similarly valuable data regarding SARS-CoV-2 key mutations as next-generation sequencing (NGS) but at 5–10 times cheaper and identified the rapid emergence of Omicron variant in Massachusetts 8 days before NGS. Five hundred twenty-five clinical samples were used to assess mCARMEN performance compared to qRT-PCR. The assay was able to identify 100% of qRT-PCR positive samples and even outperformed PCR in samples exposed to RNA degradation rendering a 100% sensitivity for mCARMEN and 88% for PCR. In addition, unlike other CRISPR assays, mCARMEN was able to identify and quantify Influenza and SARS-CoV-2 copies in samples by using a combined Cas13 and Cas12 approach (Welch et al., 2022).

## Next-Generation Sequencing

### Next-Generation Sequencing of Influenza

Numerous commonly found infectious diseases are managed without ever finding the causative pathogen. One study found that in 2,259 hospitalized patients with community-acquired pneumonia, an etiologic pathogen could be identified in only 38% of cases (Jain et al., 2015). NGS may represent the key to overcoming this limitation as it has been proven to identify bacteria, viruses, fungi, and parasites directly from clinical samples, thus allowing an accurate diagnosis. Additionally, NGS was also proven to find and characterize novel pathogens (Li et al., 2021).

Next-generation sequencing (NGS) enables the simultaneous analysis of multiple samples and can be used to detect and characterize multiple agents in a single specimen. NGS eliminates the need for prior knowledge of the viral genome and has an advantage over traditional nucleic acid detection methods that require target-specific primers (Kustin et al., 2019). Additionally, NGS identifies novel viruses in the characterization of genetic variants and offers critical information regarding mutations associated with increased virulence or treatment resistance, thus improving epidemiologic surveillance and guiding treatment decisions (Graf et al., 2016; Vemula et al., 2016; O’Flaherty et al., 2018).

Zhao et al. developed a RT-PCR-NGS platform that enabled the simultaneous detection of unknown influenza infections and coinfections in a single tube assay. Assay performance was tested using 162 influenza-positive archived clinical samples. The assay was not only able to identify the influenza virus in 99.4% of the samples but also provided accurate genotyping. NGS enabled the simultaneous identification of specific influenza subtypes and provided information regarding specific mutations such as the E627K mutation in the PB2 protein of influenza A(H3N2) linked to increased virulence the S31N mutation in the M2 protein associated with treatment resistance (Zhao et al., 2016). Furthermore, Next-generation sequencing-based metagenomics and deep sequencing strategies were proven able to detect, identify, and characterize the 2009 pandemic H1N1 influenza A virus. Using the Virochip array, Greninger et al. detected the novel virus without any prior information at concentrations close to RT-PCR detection limits. The assay also enabled the *de novo* assembly of the whole 2009 H1N1 genome using 17 clinical samples (Greninger et al., 2010).

Next-generation sequencing has several drawbacks that limit its widespread use. NGS is time-consuming and requires expensive equipment and highly trained personnel (Courtney et al., 2021). However, the main limitation hindering NGS efficiency in influenza diagnosis is the low amount of viral RNA related to host and commensal nucleic acids present in the clinical sample. To bypass this limit, target-based enrichment probes have been designed, thus providing improved NGS sensitivity in viral RNA identification and analysis (O’Flaherty et al., 2018). NGS can be accomplished with several methodologies, namely sequencing-by-ligation (SOLiD technology), sequencing-by-hybridization (resequencing microarray), and sequencing-by-synthesis (Illumina, Ion Torrent) (Slatko et al., 2018). Despite limitations, NGS is the most promising approach to Influenza genome sequencing, thus enabling new pathways in understanding treatment resistance, identifying novel therapeutics, and assuring epidemiological surveillance through the accurate identification of novel variants (Van Poelvoorde et al., 2020).

### Next-Generation Sequencing of SARS-CoV-2

In late December 2019, reports of viral pneumonia caused by an unidentified pathogen were reported in Wuhan, China. NGS was employed for the identification and characterization of the novel pathogen. NGS enabled the phylogenetic analysis, revealing that the virus belongs to the subgenus Sarbecovirus, a member of the Betacoronavirus genus. Furthermore, genomic analyses led to the revelation that although SARS-CoV-2 displays high similarities to bat-SL-CoVZC45 and bat-SL-CoVZXC21, the receptor-binding domain (S1) sequence of the spike protein (S) was more similar to that of SARS-CoV, thus suggesting that SARS-CoV-2 gains entry into cells *via* the ACE-2 receptor (Lu R. et al., 2020; Zhou et al., 2020; Zhu et al., 2020). Sequencing also demonstrated the presence of a furin-like cleavage in the spike protein of SARS-CoV-2. This cleavage site, absent in other coronaviruses, mimics the furin cleavable peptide on the epithelial sodium channel α-subunit (ENaC-α). Thus SARS-CoV-2 activates the ENaC-α causing cellular electrolyte imbalance responsible for fluid accumulation in COVID-19 patients (Anand et al., 2020; Coutard et al., 2020).

Next-generation sequencing can also be applied to the patient’s genome to underline particularities that may pertain to particular disease susceptibility. Ellinghaus et al. performed a genome-wide analysis on 1,610 patients with COVID-19 associated respiratory failure and found a higher risk for respiratory failure in patients with the rs11385942 insertion-deletion GA/A SNP at chromosome 3p21.31 and the rs657152 A/C SNP at chromosome 9q34.2. The association signal in chromosome 9q34.2 was located in the ABO blood group’s locus and demonstrated a higher risk for A-positive and a protective effect for group O. The mutated region in chromosome 3 comprises a gene encoding Sodium/Imino-acid Transporter 1 (SIT1) which interacts with ACE-2 and the CC-motif chemokine receptor 9 (CCR9) and the C-X-C motif chemokine receptor 6 (CXCR6) that regulate pulmonary CD8 memory T-cells immune response to airway pathogens (Severe Covid-19 GWAS Group et al., 2020). The GenOMICC (Genetics of Mortality in Critical Care) study performed genome-wide associations in 2,244 critically ill COVID-19 patients. They found a significant association between low expression of interferon receptor gene IFNAR2 or high expression of tyrosine kinase 2 and severe disease. Additionally, transcriptome-wide analysis revealed that monocyte-macrophage chemotactic receptor CCR2 high expression in lung tissue was also linked to severe COVID-19 disease (Pairo-Castineira et al., 2021). The same working group later performed genome sequencing on 7,491 COVID-19 patients compared to 48,400 controls and found 22 independent variants associated with a life-threatening disease form. Variants included genes involved in interferon signaling – IL10RB, PLSCR1, myeloid cells differentiation – BCL11A, TAC4, CSF2, and mucin expression -MUC1. The group also reported the protective effect of a stop-gain mutation in chromosome 19:48703417:G:A, leading to a non-secretor FUT2 phenotype (Kousathanas et al., 2021).

Next-generation sequencing may also be employed in public health surveillance and disease control. SARS-CoV-2 genetic sequencing provides data on disease origin, global transmission, and epidemiological history. A metagenomic NGS study performed on ten newly sequenced SARS-CoV-2 genomes obtained from COVID-19 patients in Hubei combined with 136 genomes from the GISAID database could trace the virus sample’s origin to the original SARS-CoV-2 haplotype found in patients living near the Huanan Seafood Wholesale Market (Chen et al., 2021). Meanwhile, Lu et al. generated 53 genomes from infected patients in the Guangdong region and demonstrated that the infections were likely to be related to travel and not local communities (Lu J. et al., 2020). Similarly, Lorruso et al. used NGS to trace infections from the Abruzzo region to a sequence originating in a sample from Northern Europe with a travel history to Italy based on the presence of the R203K and G204R mutations in the N protein (Lorusso et al., 2020).

Next-generation sequencing has been widely used to monitor the emergence of mutations and new variants (John et al., 2021). The COVID-19 Genomics United Kingdom (COG-UK) group performs periodic whole SARS-CoV-2 genome sequencing. As a result of this surveillance using NGS, they quickly identified and characterized the B.1.1.7 variant responsible for more than half of COVID-19 cases in southeast England. Genomic surveillance was also able to detect the delta variant responsible for 90% of cases in the United Kingdom. Thus, efficient genomic surveillance enables the prompt identification of new variants enabling proper public health measures and assessment of treatment efficiency (Robishaw et al., 2021). NGS also identified co-infection with other pathogens as up to 19% of COVID-19 patients may present coinfection (Moore et al., 2020; Musuuza et al., 2021). The advantages and limitations of previously discussed SARS-CoV-2 diagnostic assays are summarized in Table 2.

## Conclusion

Influenza viruses infect almost a quarter of the world population worldwide. Periodic antigenic shits are responsible for the upsurge of novel variants that elude preexisting immunity and are responsible for pandemic outbreaks. Systemic and upper respiratory symptoms dominate influenza clinical presentation. Although most infections are mild, some patients develop complications that are the leading cause of death associated with influenza. Most frequently, Influenza infections are complicated with viral or bacterial pneumonia that in some patients leads to ARDS. Influenza may also cause exacerbation of underlying respiratory or cardiac disease leading to significant morbidity. Neurologic complications are rare in adults but more frequent in pediatric patients, where they are responsible for high mortality.

SARS-CoV-2 infection most often presents with flu-like symptoms, but it may display various clinical presentations often involving gastrointestinal and neurological symptoms. However, unlike influenza, a higher percentage of cases develop severe disease with high morbidity and mortality. Across reports, up to one-third of the cases may develop ARDS. The increased and prolonged inflammatory response is responsible for late complications such as fibrosis and reduced oxygen diffusion capacity. Some degree of neurological involvement is also present in more than 80% of cases, while the prothrombotic status causes thromboembolic events in 40% of the patients.

After 2 years of an ongoing pandemic responsible for more than 6 million deaths worldwide, SARS-CoV-2 is here to stay. Despite vaccination and the ever-evolving therapeutic landscape, the high mutation rate responsible for the emergence of new variants limiting vaccine and treatment efficiency makes SARS-CoV-2 a significant public health concern. Meanwhile, lockdowns, restrictions, and the wide use of face masks limited the spread of seasonal Influenza. However, as restrictions are being lifted, influenza outbreaks will likely overlap with COVID-19, thus posing a diagnostic challenge. In this review, we covered Influenza and SARS-CoV-2 characteristics, clinical presentation while focusing on the diagnosis. RT-PCR remains the gold standard for the diagnosis of both infections. RT-PCR multiplexing panels that enable the diagnosis of Influenza and SARS-COV-2 are being developed, allowing an accurate diagnosis for patients presenting with flu-like symptoms. However, RT-PCR has several limitations. Equipment requirements and reagents cost limit their use in point-of-care settings and low-income countries. In addition, RT-PCR requires trained personnel for both sample collection and performing the procedure. Furthermore, primer selection and genetic material in clinical samples limit its accuracy. Isothermal amplification techniques like LAMP and CRISPR are being developed to answer these limitations. They promise an accurate diagnosis in a point of care setting without the need for trained personnel or expensive equipment. Rapid diagnostic tests also ease the healthcare system’s strain, allowing a quick and inexpensive population screening. Novel technologies such as NGS provide insight into the virus pathogenesis and discover novel variants offering information that may guide new therapeutics and the development of more effective vaccines.

## Author Contributions

AH, SV, and L-AP: conceptualization and writing (original draft preparation). CC, L-AP, and AH: methodology. SV, L-AP, AH, and SC: data curation. CC, SC, and L-AP: writing (review and editing) and supervision. AH and SV: visualization. AM and CC: project administration. CC, SC, and AM: funding acquisition. All authors contributed to the article and approved the submitted version.

## Conflict of Interest

The authors declare that the research was conducted in the absence of any commercial or financial relationships that could be construed as a potential conflict of interest.

## Publisher’s Note

All claims expressed in this article are solely those of the authors and do not necessarily represent those of their affiliated organizations, or those of the publisher, the editors and the reviewers. Any product that may be evaluated in this article, or claim that may be made by its manufacturer, is not guaranteed or endorsed by the publisher.

## References

[B1] Abbasi-Oshaghi E. Mirzaei F. Farahani F. Khodadadi I. Tayebinia H. (2020). Diagnosis and treatment of coronavirus disease 2019 (COVID-19): laboratory, PCR, and chest CT imaging findings. *Int. J. Surg.* 79 143–153. 10.1016/j.ijsu.2020.05.018 32422384 PMC7227548

[B2] Abe T. Segawa Y. Watanabe H. Yotoriyama T. Kai S. Yasuda A. (2011). Point-of-care testing system enabling 30 min detection of influenza genes. *Lab Chip* 11:1166. 10.1039/c0lc00519c 21311813

[B3] Ackerman C. M. Myhrvold C. Thakku S. G. Freije C. A. Metsky H. C. Yang D. K. (2020). Massively multiplexed nucleic acid detection with Cas13. *Nature* 582 277–282. 10.1038/s41586-020-2279-8 32349121 PMC7332423

[B4] Ahrberg C. D. Lee J. M. Chung B. G. (2019). Microwell array-based digital PCR for influenza virus detection. *Biochip J.* 13 269–276. 10.1007/s13206-019-3302-8

[B5] Ali Z. Aman R. Mahas A. Rao G. S. Tehseen M. Marsic T. (2020). iSCAN: an RT-LAMP-coupled CRISPR-Cas12 module for rapid, sensitive detection of SARS-CoV-2. *Virus Res.* 288:198129. 10.1016/j.virusres.2020.198129 32822689 PMC7434412

[B6] Anand P. Puranik A. Aravamudan M. Venkatakrishnan A. J. Soundararajan V. (2020). SARS-CoV-2 strategically mimics proteolytic activation of human ENaC. *Elife* 9:e58603. 10.7554/eLife.58603 32452762 PMC7343387

[B7] Anantharajah A. Helaers R. Defour J.-P. Olive N. Kabera F. Croonen L. (2021). How to choose the right real-time RT-PCR primer sets for the SARS-CoV-2 genome detection? *J. Virol. Methods* 295:114197. 10.1016/j.jviromet.2021.114197 34033854 PMC8141720

[B8] Arevalo-Rodriguez I. Buitrago-Garcia D. Simancas-Racines D. Zambrano-Achig P. Del Campo R. Ciapponi A. (2020). False-negative results of initial RT-PCR assays for COVID-19: a systematic review. *PLoS One* 15:e0242958. 10.1371/journal.pone.0242958 33301459 PMC7728293

[B9] Artesi M. Bontems S. Göbbels P. Franckh M. Maes P. Boreux R. (2020). A recurrent mutation at position 26340 of SARS-CoV-2 is associated with failure of the E gene quantitative reverse transcription-PCR utilized in a commercial dual-target diagnostic assay. *J. Clin. Microbiol.* 58 e01598–20. 10.1128/JCM.01598-20 32690547 PMC7512182

[B10] Azhar M. Phutela R. Kumar M. Ansari A. H. Rauthan R. Gulati S. (2021). Rapid and accurate nucleobase detection using FnCas9 and its application in COVID-19 diagnosis. *Biosens. Bioelectron.* 183:113207. 10.1016/j.bios.2021.113207 33866136 PMC8020606

[B11] Bai Z. Cao Y. Liu W. Li J. (2021). The SARS-CoV-2 nucleocapsid protein and its role in viral structure, biological functions, and a potential target for drug or vaccine mitigation. *Viruses* 13:1115. 10.3390/v13061115 34200602 PMC8227405

[B12] Banerjee A. Kulcsar K. Misra V. Frieman M. Mossman K. (2019). Bats and coronaviruses. *Viruses* 11:41. 10.3390/v11010041 30634396 PMC6356540

[B13] Baral N. Adhikari P. Adhikari G. Karki S. (2020). Influenza myocarditis: a literature review. *Cureus* 12:e12007. 10.7759/cureus.12007 33437555 PMC7793451

[B14] Bazdyrev E. Rusina P. Panova M. Novikov F. Grishagin I. Nebolsin V. (2021). Lung fibrosis after COVID-19: treatment prospects. *Pharmaceuticals* 14:807. 10.3390/ph14080807 34451904 PMC8398080

[B15] Bektaş A. Covington M. F. Aidelberg G. Arce A. Matute T. Núñez I. (2021). Accessible LAMP-Enabled Rapid Test (ALERT) for Detecting SARS-CoV-2. *Viruses* 13:742. 10.3390/v13050742 33922716 PMC8146324

[B16] Bennett J. E. Blaser M. J. Dolin R. (2019). *Mandell, Douglas, and Bennett’s Principles and Practice of Infectious Diseases.* Edn. 9 Philadelphia, PA: Elsevier/Saunders.

[B17] Benzigar M. R. Bhattacharjee R. Baharfar M. Liu G. (2021). Current methods for diagnosis of human coronaviruses: pros and cons. *Anal. Bioanal. Chem.* 413 2311–2330. 10.1007/s00216-020-03046-0 33219449 PMC7679240

[B18] Bikdeli B. Madhavan M. V. Jimenez D. Chuich T. Dreyfus I. Driggin E. (2020). COVID-19 and thrombotic or thromboembolic disease: implications for prevention, antithrombotic therapy, and follow-up: JACC state-of-the-art review. *J. Am. Coll. Cardiol.* 75 2950–2973. 10.1016/j.jacc.2020.04.031 32311448 PMC7164881

[B19] Boivin G. Hardy I. Tellier G. Maziade J. (2000). Predicting influenza infections during epidemics with use of a clinical case definition. *Clin. Infect. Dis.* 31 1166–1169. 10.1086/317425 11073747

[B20] Bouvier N. M. Palese P. (2008). The biology of influenza viruses. *Vaccine* 26 (Suppl. 4), D49–D53. 10.1016/j.vaccine.2008.07.039 19230160 PMC3074182

[B21] Broughton J. P. Deng X. Yu G. Fasching C. L. Servellita V. Singh J. (2020). CRISPR-Cas12-based detection of SARS-CoV-2. *Nat. Biotechnol.* 38 870–874. 10.1038/s41587-020-0513-4 32300245 PMC9107629

[B22] Brümmer L. E. Katzenschlager S. Gaeddert M. Erdmann C. Schmitz S. Bota M. (2021). Accuracy of novel antigen rapid diagnostics for SARS-CoV-2: a living systematic review and meta-analysis. *PLoS Med.* 18:e1003735. 10.1371/journal.pmed.1003735 34383750 PMC8389849

[B23] Callaway E. (2020). The coronavirus is mutating - does it matter? *Nature* 585 174–177. 10.1038/d41586-020-02544-6 32901123

[B24] Cao X. Fang K. Li Y. Zhou J. Guo X. (2022). The diagnostic accuracy of Xpert Xpress to SARS-CoV-2: a systematic review. *J. Virol. Methods* 301:114460. 10.1016/j.jviromet.2022.114460 35032480 PMC8754461

[B25] Cao Y. Liu X. Xiong L. Cai K. (2020). Imaging and clinical features of patients with 2019 novel coronavirus SARS-CoV-2: a systematic review and meta-analysis. *J. Med. Virol.* 92 1449–1459. 10.1002/jmv.25822 32242947 PMC7228215

[B26] Casiano-Colón A. E. Hulbert B. B. Mayer T. K. Walsh E. E. Falsey A. R. (2003). Lack of sensitivity of rapid antigen tests for the diagnosis of respiratory syncytial virus infection in adults. *J. Clin. Virol.* 28 169–174. 10.1016/S1386-6532(03)00002-7 12957187

[B27] Centers for Disease Control and Prevention [CDC] (2021a). *Evaluation of Rapid Influenza Diagnostic Tests for Detection of Novel Influenza A (H1N1) Virus — United States, 2009.* Available online at: https://www.cdc.gov/mmwr/preview/mmwrhtml/mm5830a2.htm (Accessed June 11, 2021) 19661856

[B28] Centers for Disease Control and Prevention [CDC] (2021b). *Information on Rapid Molecular Assays, RT-PCR, and other Molecular Assays for Diagnosis of Influenza Virus Infection | CDC.* Available online at: https://www.cdc.gov/flu/professionals/diagnosis/molecular-assays.htm (Accessed May 20, 2021)

[B29] Centers for Disease Control and Prevention [CDC] (2021c). *Rapid Influenza Diagnostic Tests | CDC.* Available online at: https://www.cdc.gov/flu/professionals/diagnosis/clinician_guidance_ridt.htm (Accessed June 10, 2021)

[B30] Centers for Disease Control and Prevention [CDC] (2022). *Interim Guidelines for COVID-19 Antibody Testing | CDC.* Available online at: https://www.cdc.gov/coronavirus/2019-ncov/lab/resources/antibody-tests-guidelines.html (Accessed February 17, 2022)

[B31] Cevik M. Tate M. Lloyd O. Maraolo A. E. Schafers J. Ho A. (2021). SARS-CoV-2, SARS-CoV, and MERS-CoV viral load dynamics, duration of viral shedding, and infectiousness: a systematic review and meta-analysis. *Lancet. Microbe* 2 e13–e22. 10.1016/S2666-5247(20)30172-5 33521734 PMC7837230

[B32] Chan L. Chaudhary K. Saha A. Chauhan K. Vaid A. Zhao S. (2021). AKI in hospitalized patients with COVID-19. *J. Am. Soc. Nephrol.* 32 151–160. 10.1681/ASN.2020050615 32883700 PMC7894657

[B33] Chen J. S. Ma E. Harrington L. B. Da Costa M. Tian X. Palefsky J. M. (2018). CRISPR-Cas12a target binding unleashes indiscriminate single-stranded DNase activity. *Science* 360 436–439. 10.1126/science.aar6245 29449511 PMC6628903

[B34] Chen X. Kang Y. Luo J. Pang K. Xu X. Wu J. (2021). Next-generation sequencing reveals the progression of COVID-19. *Front. Cell. Infect. Microbiol.* 11:632490. 10.3389/fcimb.2021.632490 33777844 PMC7991797

[B35] Cheng A. Riedel S. Arnaout R. Kirby J. E. (2022). Verification of the Abbott Alinity m Resp-4-Plex assay for detection of SARS-CoV-2, influenza A/B, and respiratory syncytial virus. *Diagn. Microbiol. Infect. Dis.* 102:115575. 10.1016/j.diagmicrobio.2021.115575 34839127 PMC8532379

[B36] Chu D. K. W. Pan Y. Cheng S. M. S. Hui K. P. Y. Krishnan P. Liu Y. (2020). Molecular diagnosis of a novel coronavirus (2019-nCoV) causing an outbreak of pneumonia. *Clin. Chem.* 66 549–555. 10.1093/clinchem/hvaa029 32031583 PMC7108203

[B37] Chung H.-Y. Jian M.-J. Chang C.-K. Lin J.-C. Yeh K.-M. Chen C.-W. (2021a). Novel dual multiplex real-time RT-PCR assays for the rapid detection of SARS-CoV-2, influenza A/B, and respiratory syncytial virus using the BD MAX open system. *Emerg. Microbes Infect.* 10 161–166. 10.1080/22221751.2021.1873073 33410371 PMC7832498

[B38] Chung H.-Y. Jian M.-J. Chang C.-K. Lin J.-C. Yeh K.-M. Yang Y.-S. (2021b). Multicenter study evaluating one multiplex RT-PCR assay to detect SARS-CoV-2, influenza A/B, and respiratory syncytia virus using the LabTurbo AIO open platform: epidemiological features, automated sample-to-result, and high-throughput testing. *Aging* 13 24931–24942. 10.18632/aging.203761 34897035 PMC8714143

[B39] Cohen M. Zhang X. Senaati H. P. Chen H. Varki N. M. Schooley R. T. (2013). Influenza A penetrates host mucus by cleaving sialic acids with neuraminidase. *Virol. J.* 10:321. 10.1186/1743-422X-10-321 24261589 PMC3842836

[B40] Corman V. M. Landt O. Kaiser M. Molenkamp R. Meijer A. Chu D. K. (2020). Detection of 2019 novel coronavirus (2019-nCoV) by real-time RT-PCR. *Eurosurveillance* 25 185–193. 10.2807/1560-7917.ES.2020.25.3.2000045 31992387 PMC6988269

[B41] Courtney S. J. Stromberg Z. R. Kubicek-Sutherland J. Z. (2021). Nucleic acid-based sensing techniques for diagnostics and surveillance of influenza. *Biosensors* 11:47. 10.3390/bios11020047 33673035 PMC7918464

[B42] Coutard B. Valle C. de Lamballerie X. Canard B. Seidah N. G. Decroly E. (2020). The spike glycoprotein of the new coronavirus 2019-nCoV contains a furin-like cleavage site absent in CoV of the same clade. *Antiviral Res.* 176:104742. 10.1016/j.antiviral.2020.104742 32057769 PMC7114094

[B43] Cox N. J. Subbarao K. (1999). Influenza. *Lancet* 354 1277–1282. 10.1016/S0140-6736(99)01241-6 10520648

[B44] da Rosa Mesquita R. Francelino Silva Junior L. C. Santos Santana F. M. Farias de Oliveira T. Campos Alcântara R. Monteiro Arnozo G. (2021). Clinical manifestations of COVID-19 in the general population: systematic review. *Wien. Klin. Wochenschr.* 133 377–382. 10.1007/s00508-020-01760-4 33242148 PMC7689634

[B45] de Groot R. J. Baker S. C. Baric R. S. Brown C. S. Drosten C. Enjuanes L. (2013). Middle East respiratory syndrome coronavirus (MERS-CoV): announcement of the Coronavirus Study Group. *J. Virol.* 87 7790–7792. 10.1128/JVI.01244-13 23678167 PMC3700179

[B46] Dong H. Wang S. Zhang J. Zhang K. Zhang F. Wang H. (2021). Structure-based primer design minimizes the risk of PCR failure caused by SARS-CoV-2 mutations. *Front. Cell. Infect. Microbiol.* 11:741147. 10.3389/fcimb.2021.741147 34760717 PMC8573093

[B47] Dong H. Zhang Y. Xiong H. Yan A. Ding G. Chen Y. (2010). Detection of human novel influenza A (H1N1) viruses using multi-fluorescent real-time RT-PCR. *Virus Res.* 147 85–90. 10.1016/j.virusres.2009.10.011 19883704

[B48] Drinka P. J. Krause P. Nest L. (2003). Clinical features of influenza a virus infection in older hospitalized persons. *J. Am. Geriatr. Soc.* 51 1184–1184. 10.1046/j.1532-5415.2003.51374.x 12890092

[B49] Dzia̧bowska K. Czaczyk E. Nidzworski D. (2018). Detection methods of human and animal influenza virus—current trends. *Biosensors* 8:94. 10.3390/bios8040094 30340339 PMC6315519

[B50] Eigner U. Reucher S. Hefner N. Staffa-Peichl S. Kolb M. Betz U. (2019). Clinical evaluation of multiplex RT-PCR assays for the detection of influenza A/B and respiratory syncytial virus using a high throughput system. *J. Virol. Methods* 269 49–54. 10.1016/j.jviromet.2019.03.015 30946852

[B51] European Centre for Disease Prevention and Control [ECDC] (2020). *European Centre for Disease Prevention and Control. Reinfection with SARS-CoV: Considerations for Public Health Response.* Solna: ECDC.

[B52] Fakheran O. Dehghannejad M. Khademi A. (2020). Saliva as a diagnostic specimen for detection of SARS-CoV-2 in suspected patients: a scoping review. *Infect. Dis. poverty* 9:100. 10.1186/s40249-020-00728-w 32698862 PMC7374661

[B53] Falzone L. Gattuso G. Tsatsakis A. Spandidos D. A. Libra M. (2021). Current and innovative methods for the diagnosis of COVID-19 infection (Review). *Int. J. Mol. Med.* 47:100. 10.3892/ijmm.2021.4933 33846767 PMC8043662

[B54] Fanelli V. Fiorentino M. Cantaluppi V. Gesualdo L. Stallone G. Ronco C. (2020). Acute kidney injury in SARS-CoV-2 infected patients. *Crit. Care* 24:155. 10.1186/s13054-020-02872-z 32299479 PMC7161433

[B55] Favas T. T. Dev P. Chaurasia R. N. Chakravarty K. Mishra R. Joshi D. (2020). Neurological manifestations of COVID-19: a systematic review and meta-analysis of proportions. *Neurol. Sci.* 41 3437–3470. 10.1007/s10072-020-04801-y 33089477 PMC7577367

[B56] Feng H. Liu Y. Lv M. Zhong J. (2020). A case report of COVID-19 with false negative RT-PCR test: necessity of chest CT. *Jpn. J. Radiol.* 38 409–410. 10.1007/s11604-020-00967-9 32266524 PMC7136155

[B57] Food and Drug Administration [FDA] (2021). *SARS-CoV-2 Viral Mutations: Impact on COVID-19 Tests On this page: Genetic Variations: Background and Considerations General Information for Clinical Laboratory Staff and Healthcare Providers. U.S. Food Drug Adm., 1–6.* Silver Spring: Food and Drug Administration.

[B58] Food and Drug Administration [FDA] (2022). *In Vitro Diagnostics EUAs - Antigen Diagnostic Tests for SARS-CoV-2 | FDA.* Available online at: https://www.fda.gov/medical-devices/coronavirus-disease-2019-covid-19-emergency-use-authorizations-medical-devices/in-vitro-diagnostics-euas-antigen-diagnostic-tests-sars-cov-2 (Accessed February 17, 2022)

[B59] Freije C. A. Myhrvold C. Boehm C. K. Lin A. E. Welch N. L. Carter A. (2019). Programmable Inhibition and Detection of RNA viruses using Cas13. *Mol. Cell* 76 826–837.e11. 10.1016/j.molcel.2019.09.013 31607545 PMC7422627

[B60] Gavin P. J. Thomson R. B. (2004). Review of rapid diagnostic tests for influenza. *Clin. Appl. Immunol. Rev.* 4 151–172. 10.1016/S1529-1049(03)00064-3

[B61] Gibson P. G. Qin L. Puah S. H. (2020). COVID-19 acute respiratory distress syndrome (ARDS): clinical features and differences from typical pre-COVID-19 ARDS. *Med. J. Aust.* 213 54–56.e1. 10.5694/mja2.50674 32572965 PMC7361309

[B62] Graf E. H. Simmon K. E. Tardif K. D. Hymas W. Flygare S. Eilbeck K. (2016). Unbiased detection of respiratory viruses by use of RNA sequencing-based metagenomics: a systematic comparison to a commercial PCR panel. *J. Clin. Microbiol.* 54 1000–1007. 10.1128/JCM.03060-15 26818672 PMC4809917

[B63] Green D. A. StGeorge K. (2018). Rapid antigen tests for influenza: rationale and significance of the FDA reclassification. *J. Clin. Microbiol.* 56:e00711-18. 10.1128/JCM.00711-18 29899007 PMC6156320

[B64] Greninger A. L. Chen E. C. Sittler T. Scheinerman A. Roubinian N. Yu G. (2010). A metagenomic analysis of pandemic influenza A (2009 H1N1) infection in patients from North America. *PLoS One* 5:e13381. 10.1371/journal.pone.0013381 20976137 PMC2956640

[B65] Gupta R. Kazi T. A. Dey D. Ghosh A. Ravichandiran V. Swarnakar S. (2021). CRISPR detectives against SARS-CoV-2: a major setback against COVID-19 blowout. *Appl. Microbiol. Biotechnol.* 105 7593–7605. 10.1007/s00253-021-11583-6 34542686 PMC8450312

[B66] Hansen G. Marino J. Wang Z.-X. Beavis K. G. Rodrigo J. Labog K. (2021). Clinical performance of the point-of-care cobas liat for detection of SARS-CoV-2 in 20 minutes: a multicenter study. *J. Clin. Microbiol.* 59 e02811–20. 10.1128/JCM.02811-20 33239382 PMC8111162

[B67] Harapan B. N. Yoo H. J. (2021). Neurological symptoms, manifestations, and complications associated with severe acute respiratory syndrome coronavirus 2 (SARS-CoV-2) and coronavirus disease 19 (COVID-19). *J. Neurol.* 268 3059–3071. 10.1007/s00415-021-10406-y 33486564 PMC7826147

[B68] Hasan M. R. Sundararaju S. Manickam C. Mirza F. Al-Hail H. Lorenz S. (2021). A novel point mutation in the N gene of SARS-CoV-2 may affect the detection of the virus by reverse transcription-quantitative PCR. *J. Clin. Microbiol.* 59 19–21. 10.1128/JCM.03278-20 33472904 PMC8092750

[B69] Horai Y. Miyamura T. Takahama S. Sonomoto K. Nakamura M. Ando H. (2010). Influenza virus B-associated hemophagocytic syndrome and recurrent pericarditis in a patient with systemic lupus erythematosus. *Mod. Rheumatol.* 20 178–182. 10.1007/s10165-009-0241-6 19898920

[B70] Hou H. Wang T. Zhang B. Luo Y. Mao L. Wang F. (2020). Detection of IgM and IgG antibodies in patients with coronavirus disease 2019. *Clin. Transl. Immunol.* 9:e01136. 10.1002/cti2.1136 32382418 PMC7202656

[B71] Huang C. Wen T. Shi F.-J. Zeng X.-Y. Jiao Y.-J. (2020). Rapid detection of IgM antibodies against the SARS-CoV-2 virus *via* colloidal gold nanoparticle-based lateral-flow assay. *ACS Omega* 5 12550–12556. 10.1021/acsomega.0c01554 32542208 PMC7241732

[B72] Jackson C. B. Farzan M. Chen B. Choe H. (2022). Mechanisms of SARS-CoV-2 entry into cells. *Nat. Rev. Mol. Cell Biol.* 23 3–20. 10.1038/s41580-021-00418-x 34611326 PMC8491763

[B73] Jain S. Self W. H. Wunderink R. G. Fakhran S. Balk R. Bramley A. M. (2015). Community-acquired pneumonia requiring hospitalization among U.S. *Adults. N. Engl. J. Med.* 373 415–427. 10.1056/NEJMoa1500245 26172429 PMC4728150

[B74] Jayamohan H. Lambert C. J. Sant H. J. Jafek A. Patel D. Feng H. (2021). SARS-CoV-2 pandemic: a review of molecular diagnostic tools including sample collection and commercial response with associated advantages and limitations. *Anal. Bioanal. Chem.* 413 49–71. 10.1007/s00216-020-02958-1 33073312 PMC7568947

[B75] John G. Sahajpal N. S. Mondal A. K. Ananth S. Williams C. Chaubey A. (2021). Next-Generation Sequencing (NGS) in COVID-19: a tool for SARS-CoV-2 diagnosis, monitoring new strains and phylodynamic modeling in molecular epidemiology. *Curr. Issues Mol. Biol.* 43 845–867. 10.3390/cimb43020061 34449545 PMC8929009

[B76] Kevadiya B. D. Machhi J. Herskovitz J. Oleynikov M. D. Blomberg W. R. Bajwa N. (2021). Diagnostics for SARS-CoV-2 infections. *Nat. Mater.* 20 593–605. 10.1038/s41563-020-00906-z 33589798 PMC8264308

[B77] Killingley B. Nguyen-Van-Tam J. (2013). Routes of influenza transmission. *Influenza Other Respir. Viruses* 7 42–51. 10.1111/irv.12080 24034483 PMC5909391

[B78] Kim T. Y. Kim J.-Y. Shim H. J. Yun S. A. Jang J. Huh H. J. (2021). Comparison of the PowerChek SARS-CoV-2, Influenza A&B, RSV multiplex real-time PCR Kit and BioFire Respiratory Panel 2.1 for simultaneous detection of SARS-CoV-2, influenza A and B, and respiratory syncytial virus. *J. Virol. Methods* 298:114304. 10.1016/j.jviromet.2021.114304 34592335 PMC8482523

[B79] Kim T. Y. Kim J.-Y. Shim H. J. Yun S. A. Jang J. Huh H. J. (2022). Performance evaluation of the PowerChek SARS-CoV-2, Influenza A & B Multiplex Real-Time PCR Kit in Comparison with the BioFire respiratory panel. *Ann. Lab. Med.* 42 473–477. 10.3343/alm.2022.42.4.473 35177568 PMC8859561

[B80] Kirtipal N. Bharadwaj S. Kang S. G. (2020). From SARS to SARS-CoV-2, insights on structure, pathogenicity and immunity aspects of pandemic human coronaviruses. *Infect. Genet. Evol.* 85:104502. 10.1016/j.meegid.2020.104502 32798769 PMC7425554

[B81] Klein E. Y. Monteforte B. Gupta A. Jiang W. May L. Hsieh Y.-H. (2016). The frequency of influenza and bacterial coinfection: a systematic review and meta-analysis. *Influenza Other Respir. Viruses* 10 394–403. 10.1111/irv.12398 27232677 PMC4947938

[B82] Kodama M. (2010). Influenza myocarditis. *Circ. J.* 74 2060–2061. 10.1253/circj.cj-10-0833 20838004

[B83] Kousathanas A. Pairo-Castineira E. Rawlik K. Stuckey A. Odhams C. A. Walker S. (2021). Whole genome sequencing identifies multiple loci for critical illness caused by COVID-19. *medRxiv [Preprint]* 10.1101/2021.09.02.21262965

[B84] Krammer F. Smith G. J. D. Fouchier R. A. M. Peiris M. Kedzierska K. Doherty P. C. (2018). Influenza. *Nat. Rev. Dis. Prim.* 4:3. 10.1038/s41572-018-0002-y 29955068 PMC7097467

[B85] Kubo T. Agoh M. Mai L. Q. Fukushima K. Nishimura H. Yamaguchi A. (2010). Development of a reverse transcription-loop-mediated isothermal amplification assay for detection of pandemic (H1N1) 2009 virus as a novel molecular method for diagnosis of pandemic influenza in resource-limited settings. *J. Clin. Microbiol.* 48 728–735. 10.1128/JCM.01481-09 20071551 PMC2832456

[B86] Kucirka L. M. Lauer S. A. Laeyendecker O. Boon D. Lessler J. (2020). Variation in false-negative rate of reverse transcriptase polymerase chain reaction-based SARS-CoV-2 tests by time since exposure. *Ann. Intern. Med.* 173 262–267. 10.7326/M20-1495 32422057 PMC7240870

[B87] Kunutsor S. K. Laukkanen J. A. (2020). Renal complications in COVID-19: a systematic review and meta-analysis. *Ann. Med.* 52 345–353. 10.1080/07853890.2020.1790643 32643418 PMC7877945

[B88] Kustin T. Ling G. Sharabi S. Ram D. Friedman N. Zuckerman N. (2019). A method to identify respiratory virus infections in clinical samples using next-generation sequencing. *Sci. Rep.* 9:2606. 10.1038/s41598-018-37483-w 30796243 PMC6384955

[B89] Lai C. K. C. Lam W. (2021). Laboratory testing for the diagnosis of COVID-19. *Biochem. Biophys. Res. Commun.* 538 226–230. 10.1016/j.bbrc.2020.10.069 33139015 PMC7598306

[B90] Lee D. Chu C.-H. Sarioglu A. F. (2021). Point-of-care toolkit for multiplex molecular diagnosis of SARS-CoV-2 and influenza A and B viruses. *ACS Sensors* 6 3204–3213. 10.1021/acssensors.1c00702 34523904

[B91] Lee N. Hui D. Wu A. Chan P. Cameron P. Joynt G. M. (2003). A major outbreak of severe acute respiratory syndrome in Hong Kong. *N. Engl. J. Med.* 348 1986–1994. 10.1056/NEJMoa030685 12682352

[B92] Leung W. K. To K.-F. Chan P. K. S. Chan H. L. Y. Wu A. K. L. Lee N. (2003). Enteric involvement of severe acute respiratory syndrome-associated coronavirus infection. *Gastroenterology* 125 1011–1017. 10.1016/s0016-5085(03)01215-0 14517783 PMC7126982

[B93] Li K. Brownley A. (2010). “Primer design for RT-PCR,” in *Methods in Molecular Biology (Clifton, N.J.) Methods in Molecular Biology*, ed. King N. (Totowa, NJ: Humana Press), 271–299. 10.1007/978-1-60761-629-0_18 20301004

[B94] Li N. Cai Q. Miao Q. Song Z. Fang Y. Hu B. (2021). High-throughput metagenomics for identification of pathogens in the clinical settings. *Small Methods* 5:2000792. 10.1002/smtd.202000792 33614906 PMC7883231

[B95] Liu R. Sheng Z. Huang C. Wang D. Li F. (2020). Influenza D virus. *Curr. Opin. Virol.* 44 154–161. 10.1016/j.coviro.2020.08.004 32932215 PMC7755673

[B96] Liu S. Pan C. (2020). Differentiating diagnosis of COVID-19 or influenza in patients based on laboratory data during flu season. *EClinicalMedicine* 26:100511. 10.1016/j.eclinm.2020.100511 32864591 PMC7445122

[B97] Loeffelholz M. J. Alland D. Butler-Wu S. M. Pandey U. Perno C. F. Nava A. (2020). Multicenter evaluation of the cepheid xpert Xpress SARS-CoV-2 Test. *J. Clin. Microbiol.* 58 e00926–20. 10.1128/JCM.00926-20 32366669 PMC7383535

[B98] Long B. Brady W. J. Koyfman A. Gottlieb M. (2020). Cardiovascular complications in COVID-19. *Am. J. Emerg. Med.* 38 1504–1507. 10.1016/j.ajem.2020.04.048 32317203 PMC7165109

[B99] Long Q.-X. Liu B.-Z. Deng H.-J. Wu G.-C. Deng K. Chen Y.-K. (2020). Antibody responses to SARS-CoV-2 in patients with COVID-19. *Nat. Med.* 26 845–848. 10.1038/s41591-020-0897-1 32350462

[B100] Lorusso A. Calistri P. Mercante M. T. Monaco F. Portanti O. Marcacci M. (2020). A “One-Health” approach for diagnosis and molecular characterization of SARS-CoV-2 in Italy. *One Heal.* 10:100135. 10.1016/j.onehlt.2020.100135 32313828 PMC7166304

[B101] Lu J. du Plessis L. Liu Z. Hill V. Kang M. Lin H. (2020). Genomic epidemiology of SARS-CoV-2 in guangdong province, China. *Cell* 181 997–1003.e9. 10.1016/j.cell.2020.04.023 32359424 PMC7192124

[B102] Lu R. Zhao X. Li J. Niu P. Yang B. Wu H. (2020). Genomic characterisation and epidemiology of 2019 novel coronavirus: implications for virus origins and receptor binding. *Lancet* 395 565–574. 10.1016/S0140-6736(20)30251-8 32007145 PMC7159086

[B103] Mahalingam S. Peter J. Xu Z. Bordoloi D. Ho M. Kalyanaraman V. S. (2021). Landscape of humoral immune responses against SARS-CoV-2 in patients with COVID-19 disease and the value of antibody testing. *Heliyon* 7:e06836. 10.1016/j.heliyon.2021.e06836 33898857 PMC8052472

[B104] Mahony J. Chong S. Bulir D. Ruyter A. Mwawasi K. Waltho D. (2013). Multiplex loop-mediated isothermal amplification (M-LAMP) assay for the detection of influenza A/H1, A/H3 and influenza B can provide a specimen-to-result diagnosis in 40min with single genome copy sensitivity. *J. Clin. Virol.* 58 127–131. 10.1016/j.jcv.2013.06.006 23827787

[B105] Mancini F. Barbanti F. Scaturro M. Fontana S. Di Martino A. Marsili G. (2021). Multiplex real-time reverse-transcription polymerase chain reaction assays for diagnostic testing of severe acute respiratory syndrome coronavirus 2 and seasonal influenza viruses: a challenge of the phase 3 pandemic setting. *J. Infect. Dis.* 223 765–774. 10.1093/infdis/jiaa658 33080031 PMC7665649

[B106] Mardian Y. Kosasih H. Karyana M. Neal A. Lau C.-Y. (2021). Review of current COVID-19 diagnostics and opportunities for further development. *Front. Med.* 8:615099. 10.3389/fmed.2021.615099 34026773 PMC8138031

[B107] Mboumba Bouassa R.-S. Tonen-Wolyec S. Veyer D. Péré H. Bélec L. (2022). Analytical performances of the AMPLIQUICK^®^ Respiratory Triplex assay for simultaneous detection and differentiation of SARS-CoV-2, influenza A/B and respiratory syncytial viruses in respiratory specimens. *PLoS One* 17:e0262258. 10.1371/journal.pone.0262258 34986156 PMC8730414

[B108] McMullen A. R. Anderson N. W. Burnham C.-A. D. (2016). Pathology consultation on influenza diagnostics. *Am. J. Clin. Pathol.* 145 440–448. 10.1093/ajcp/aqw039 27124947 PMC7110119

[B109] Migliaccio M. G. Di Mauro M. Ricciolino R. Spiniello G. Carfora V. Verde N. (2021). Renal involvement in COVID-19: a review of the literature. *Infect. Drug Resist.* 14 895–903. 10.2147/IDR.S288869 33707958 PMC7943324

[B110] Minami T. Iwata Y. Wada T. (2020). Renal complications in coronavirus disease 2019: a systematic review. *Inflamm. Regen.* 40:31. 10.1186/s41232-020-00140-9 33317643 PMC7735801

[B111] Mollaei H. R. Afshar A. A. Kalantar-Neyestanaki D. Fazlalipour M. Aflatoonian B. (2020). Comparison five primer sets from different genome region of COVID-19 for detection of virus infection by conventional RT-PCR. *Iran. J. Microbiol.* 12 185–193. 10.18502/ijm.v12i3.3234 32685113 PMC7340604

[B112] Monto A. S. Gravenstein S. Elliott M. Colopy M. Schweinle J. (2000). Clinical signs and symptoms predicting influenza infection. *Arch. Intern. Med.* 160:3243. 10.1001/archinte.160.21.3243 11088084

[B113] Moore S. C. Penrice-Randal R. Alruwaili M. Randle N. Armstrong S. Hartley C. (2020). Amplicon-based detection and sequencing of SARS-CoV-2 in nasopharyngeal swabs from patients With COVID-19 and identification of deletions in the viral genome that encode proteins involved in interferon antagonism. *Viruses* 12:1164. 10.3390/v12101164 33066701 PMC7602519

[B114] Mouliou D. S. Gourgoulianis K. I. (2021). False-positive and false-negative COVID-19 cases: respiratory prevention and management strategies, vaccination, and further perspectives. *Expert Rev. Respir. Med.* 15 993–1002. 10.1080/17476348.2021.1917389 33896332 PMC8074645

[B115] Musuuza J. S. Watson L. Parmasad V. Putman-Buehler N. Christensen L. Safdar N. (2021). Prevalence and outcomes of co-infection and superinfection with SARS-CoV-2 and other pathogens: a systematic review and meta-analysis. *PLoS One* 16:e0251170. 10.1371/journal.pone.0251170 33956882 PMC8101968

[B116] Nalla A. K. Casto A. M. Huang M.-L. W. Perchetti G. A. Sampoleo R. Shrestha L. (2020). Comparative performance of SARS-CoV-2 detection assays using seven different primer-probe sets and one assay kit. *J. Clin. Microbiol.* 58:e00557-20. 10.1128/JCM.00557-20 32269100 PMC7269385

[B117] Newton D. W. Treanor J. J. Menegus M. A. (2000). Clinical and laboratory diagnosis of influenza virus infections. *Am. J. Manag. Care* 6 S265–S275. 10977473

[B118] Ni M. Xu H. Luo J. Liu W. Zhou D. (2021). Simultaneous detection and differentiation of SARS-CoV-2, influenza A virus and influenza B virus by one-step quadruplex real-time RT-PCR in patients with clinical manifestations. *Int. J. Infect. Dis.* 103 517–524. 10.1016/j.ijid.2020.12.027 33326873 PMC7836965

[B119] Nie Q.-H. Luo X.-D. Zhang J.-Z. Su Q. (2003). Current status of severe acute respiratory syndrome in China. *World J. Gastroenterol.* 9 1635–1645. 10.3748/wjg.v9.i8.1635 12918094 PMC4611517

[B120] Nikitin N. Petrova E. Trifonova E. Karpova O. (2014). Influenza virus aerosols in the air and their infectiousness. *Adv. Virol.* 2014 859090. 10.1155/2014/859090 25197278 PMC4147198

[B121] Nörz D. Hoffmann A. Aepfelbacher M. Pfefferle S. Lütgehetmann M. (2021). Clinical evaluation of a fully automated, laboratory-developed multiplex RT-PCR assay integrating dual-target SARS-CoV-2 and influenza A/B detection on a high-throughput platform. *J. Med. Microbiol.* 70:001295. 10.1099/jmm.0.001295 33404401 PMC8131019

[B122] O’Flaherty B. M. Li Y. Tao Y. Paden C. R. Queen K. Zhang J. (2018). Comprehensive viral enrichment enables sensitive respiratory virus genomic identification and analysis by next generation sequencing. *Genome Res.* 28 869–877. 10.1101/gr.226316.117 29703817 PMC5991510

[B123] Pabbaraju K. Wong A. A. Ma R. Zelyas N. Tipples G. A. (2021). Development and validation of a multiplex reverse transcriptase-PCR assay for simultaneous testing of influenza A, influenza B and SARS-CoV-2. *J. Virol. Methods* 293:114151. 10.1016/j.jviromet.2021.114151 33839186 PMC8028604

[B124] Padoan A. Sciacovelli L. Basso D. Negrini D. Zuin S. Cosma C. (2020). IgA-Ab response to spike glycoprotein of SARS-CoV-2 in patients with COVID-19: a longitudinal study. *Clin. Chim. Acta* 507 164–166. 10.1016/j.cca.2020.04.026 32343948 PMC7194886

[B125] Pairo-Castineira E. Clohisey S. Klaric L. Bretherick A. D. Rawlik K. Pasko D. (2021). Genetic mechanisms of critical illness in COVID-19. *Nature* 591 92–98. 10.1038/s41586-020-03065-y 33307546

[B126] Paradis S. Lockamy E. Cooper C. K. Young S. (2021). Clinical evaluation of the molecular-based BD SARS-CoV-2/Flu for the BD MAXTM system. *J. Clin. Virol.* 143:104946. 10.1016/j.jcv.2021.104946 34507269 PMC8376527

[B127] Parolo C. de la Escosura-Muñiz A. Merkoçi A. (2013). Enhanced lateral flow immunoassay using gold nanoparticles loaded with enzymes. *Biosens. Bioelectron.* 40 412–416. 10.1016/j.bios.2012.06.049 22795532

[B128] Patchsung M. Jantarug K. Pattama A. Aphicho K. Suraritdechachai S. Meesawat P. (2020). Clinical validation of a Cas13-based assay for the detection of SARS-CoV-2 RNA. *Nat. Biomed. Eng.* 4 1140–1149. 10.1038/s41551-020-00603-x 32848209

[B129] Paules C. Subbarao K. (2017). Influenza. *Lancet* 390 697–708. 10.1016/S0140-6736(17)30129-0 28302313

[B130] Peaper D. R. Landry M. L. (2014). Rapid diagnosis of influenza: state of the art. *Clin. Lab. Med.* 34 365–385. 10.1016/j.cll.2014.02.009 24856533 PMC7172071

[B131] Pene F. Merlat A. Vabret A. Rozenberg F. Buzyn A. Dreyfus F. (2003). Coronavirus 229E-related pneumonia in immunocompromised patients. *Clin. Infect. Dis.* 37 929–932. 10.1086/377612 13130404 PMC7107892

[B132] Perlman S. Netland J. (2009). Coronaviruses post-SARS: update on replication and pathogenesis. *Nat. Rev. Microbiol.* 7 439–450. 10.1038/nrmicro2147 19430490 PMC2830095

[B133] Petric M. Comanor L. Petti C. A. (2006). Role of the laboratory in diagnosis of influenza during seasonal epidemics and potential pandemics. *J. Infect. Dis.* 194 S98–S110. 10.1086/507554 17163396

[B134] Petrova V. N. Russell C. A. (2018). The evolution of seasonal influenza viruses. *Nat. Rev. Microbiol.* 16 47–60. 10.1038/nrmicro.2017.118 29081496

[B135] Pisanic N. Randad P. R. Kruczynski K. Manabe Y. C. Thomas D. L. Pekosz A. (2020). COVID-19 Serology at Population Scale: SARS-CoV-2-Specific Antibody Responses in Saliva. *J. Clin. Microbiol.* 59 e02204–20. 10.1128/JCM.02204-20 33067270 PMC7771435

[B136] Pu R. Liu S. Ren X. Shi D. Ba Y. Huo Y. (2022). The screening value of RT-LAMP and RT-PCR in the diagnosis of COVID-19: systematic review and meta-analysis. *J. Virol. Methods* 300:114392. 10.1016/j.jviromet.2021.114392 34856308 PMC8629515

[B137] Rapuntean S. (2019). *Special Veterinary Virology.* Cluj-Napoca: AcademicPres Publishing House.

[B138] Rawson T. M. Moore L. S. P. Zhu N. Ranganathan N. Skolimowska K. Gilchrist M. (2020). Bacterial and fungal coinfection in individuals with coronavirus: a rapid review to support COVID-19 antimicrobial prescribing. *Clin. Infect. Dis.* 71 2459–2468. 10.1093/cid/ciaa530 32358954 PMC7197596

[B139] Robishaw J. D. Alter S. M. Solano J. J. Shih R. D. DeMets D. L. Maki D. G. (2021). Genomic surveillance to combat COVID-19: challenges and opportunities. *Lancet Microbe* 2 e481–e484. 10.1016/S2666-5247(21)00121-X 34337584 PMC8315763

[B140] Rohde G. Wiethege A. Borg I. Kauth M. Bauer T. T. Gillissen A. (2003). Respiratory viruses in exacerbations of chronic obstructive pulmonary disease requiring hospitalisation: a case-control study. *Thorax* 58 37–42. 10.1136/thorax.58.1.37 12511718 PMC1746460

[B141] Rothberg M. B. Haessler S. D. Brown R. B. (2008). Complications of viral influenza. *Am. J. Med.* 121 258–264. 10.1016/j.amjmed.2007.10.040 18374680 PMC7172971

[B142] Ruest A. Michaud S. Deslandes S. Frost E. H. (2003). Comparison of the directigen Flu A+B Test, the QuickVue influenza test, and clinical case definition to viral culture and reverse transcription-PCR for rapid diagnosis of influenza virus infection. *J. Clin. Microbiol.* 41 3487–3493. 10.1128/JCM.41.8.3487-3493.2003 12904343 PMC179849

[B143] Safiabadi Tali S. H. LeBlanc J. J. Sadiq Z. Oyewunmi O. D. Camargo C. Nikpour B. (2021). Tools and techniques for severe acute respiratory syndrome coronavirus 2 (SARS-CoV-2)/COVID-19 detection. *Clin. Microbiol. Rev.* 34 1–63. 10.1128/CMR.00228-20 33980687 PMC8142517

[B144] Sakai-Tagawa Y. Ozawa M. Tamura D. Le M. T. Q. Nidom C. A. Sugaya N. (2010). Sensitivity of influenza rapid diagnostic tests to H5N1 and 2009 pandemic H1N1 viruses. *J. Clin. Microbiol.* 48 2872–2877. 10.1128/JCM.00439-10 20554831 PMC2916590

[B145] Sellers S. A. Hagan R. S. Hayden F. G. Fischer W. A. (2017). The hidden burden of influenza: a review of the extra-pulmonary complications of influenza infection. *Influenza Other Respir. Viruses* 11 372–393. 10.1111/irv.12470 28745014 PMC5596521

[B146] Severe Covid-19 GWAS Group Ellinghaus D. Degenhardt F. Bujanda L. Buti M. Albillos A. (2020). Genomewide association study of severe covid-19 with respiratory failure. *N. Engl. J. Med.* 383 1522–1534. 10.1056/NEJMoa2020283 32558485 PMC7315890

[B147] Shaw M. W. Xu X. Li Y. Normand S. Ueki R. T. Kunimoto G. Y. (2002). Reappearance and global spread of variants of influenza B/Victoria/2/87 lineage viruses in the 2000–2001 and 2001–2002 seasons. *Virology* 303 1–8. 10.1006/viro.2002.1719 12482653

[B148] Sheridan C. (2020). Coronavirus and the race to distribute reliable diagnostics. *Nat. Biotechnol.* 38 382–384. 10.1038/d41587-020-00002-2 32265548

[B149] Shu B. Kirby M. K. Davis W. G. Warnes C. Liddell J. Liu J. (2021). Multiplex real-time reverse transcription PCR for influenza A virus, influenza B Virus, and severe acute respiratory syndrome coronavirus 2. *Emerg. Infect. Dis.* 27 1821–1830. 10.3201/eid2707.210462 34152951 PMC8237866

[B150] Siqueira J. D. Goes L. R. Alves B. M. da Silva A. C. P. de Carvalho P. S. Cicala C. (2021). Distinguishing SARS-CoV-2 bonafide re-infection from pre-existing minor variant reactivation. *Infect. Genet. Evol.* 90:104772. 10.1016/J.MEEGID.2021.104772 33592317 PMC7882217

[B151] Slatko B. E. Gardner A. F. Ausubel F. M. (2018). Overview of next-generation sequencing technologies. *Curr. Protoc. Mol. Biol.* 122 1–15. 10.1002/cpmb.59 29851291 PMC6020069

[B152] Smith C. J. Osborn A. M. (2009). Advantages and limitations of quantitative PCR (Q-PCR)-based approaches in microbial ecology. *FEMS Microbiol. Ecol.* 67 6–20. 10.1111/j.1574-6941.2008.00629.x 19120456

[B153] Song X. Delaney M. Shah R. K. Campos J. M. Wessel D. L. DeBiasi R. L. (2020). Comparison of clinical features of COVID-19 vs seasonal influenza A and B in US children. *JAMA Netw. Open* 3:e2020495. 10.1001/jamanetworkopen.2020.20495 32897374 PMC7489826

[B154] Spoto S. Valeriani E. Locorriere L. Anguissola G. B. Pantano A. L. Terracciani F. (2019). Influenza B virus infection complicated by life-threatening pericarditis: a unique case-report and literature review. *BMC Infect. Dis.* 19:40. 10.1186/s12879-018-3606-7 30630424 PMC6327550

[B155] Studahl M. (2003). Influenza virus and CNS manifestations. *J. Clin. Virol.* 28 225–232. 10.1016/S1386-6532(03)00119-7 14522059

[B156] Suo T. Liu X. Feng J. Guo M. Hu W. Guo D. (2020). ddPCR: a more accurate tool for SARS-CoV-2 detection in low viral load specimens. *Emerg. Microbes Infect.* 9 1259–1268. 10.1080/22221751.2020.1772678 32438868 PMC7448897

[B157] Tang X. Musa S. S. Zhao S. He D. (2021). Reinfection or reactivation of severe acute respiratory syndrome coronavirus 2: a systematic review. *Front. Public Heal.* 9:663045. 10.3389/fpubh.2021.663045 34178920 PMC8226004

[B158] Teymouri M. Mollazadeh S. Mortazavi H. Naderi Ghale-Noie Z. Keyvani V. Aghababaei F. (2021). Recent advances and challenges of RT-PCR tests for the diagnosis of COVID-19. *Pathol. Res. Pract.* 221:153443. 10.1016/j.prp.2021.153443 33930607 PMC8045416

[B159] To K. F. Chan P. K. Chan K. F. Lee W. K. Lam W. Y. Wong K. F. (2001). Pathology of fatal human infection associated with avian influenza A H5N1 virus. *J. Med. Virol.* 63 242–246. 10.1002/1096-9071(200103)63:3<242::aid-jmv1007<3.0.co;2-n 11170064

[B160] To K. F. Tong J. H. M. Chan P. K. S. Au F. W. L. Chim S. S. C. Chan K. C. A. (2004). Tissue and cellular tropism of the coronavirus associated with severe acute respiratory syndrome: an in-situ hybridization study of fatal cases. *J. Pathol.* 202 157–163. 10.1002/path.1510 14743497 PMC7167900

[B161] Tombuloglu H. Sabit H. Al-Khallaf H. Kabanja J. H. Alsaeed M. Al-Saleh N. (2022). Multiplex real-time RT-PCR method for the diagnosis of SARS-CoV-2 by targeting viral N, RdRP and human RP genes. *Sci. Rep.* 12:2853. 10.1038/s41598-022-06977-z 35181721 PMC8857243

[B162] Tong S. Zhu X. Li Y. Shi M. Zhang J. Bourgeois M. (2013). New world bats harbor diverse influenza A viruses. *PLoS Pathog.* 9:e1003657. 10.1371/journal.ppat.1003657 24130481 PMC3794996

[B163] Trick A. Y. Chen F.-E. Chen L. Lee P.-W. Hasnain A. C. Mostafa H. H. (2021). Magnetofluidic platform for rapid multiplexed screening of SARS-CoV-2 variants and respiratory pathogens. *medRxiv [Preprint].* 10.1101/2021.05.10.21256995 35441089 PMC9011450

[B164] Tsai J. P. Baker A. J. (2013). Influenza-associated neurological complications. *Neurocrit. Care* 18 118–130. 10.1007/s12028-012-9796-8 23138546

[B165] Tuan J. Spichler-Moffarah A. Ogbuagu O. (2021). A new positive SARS-CoV-2 test months after severe COVID-19 illness: reinfection or intermittent viral shedding? *BMJ Case Rep.* 14:e240531. 10.1136/bcr-2020-240531 33542020 PMC8098910

[B166] Uyeki T. M. Bernstein H. H. Bradley J. S. Englund J. A. File T. M. Fry A. M. (2019). Clinical practice guidelines by the infectious diseases society of America: 2018 update on diagnosis, treatment, chemoprophylaxis, and institutional outbreak management of seasonal influenzaa. *Clin. Infect. Dis.* 68 e1–e47. 10.1093/cid/ciy866 30566567 PMC6653685

[B167] Valencia D. N. (2020). Brief review on COVID-19: the 2020 pandemic caused by SARS-CoV-2. *Cureus* 12:e7386. 10.7759/cureus.7386 32337113 PMC7179986

[B168] van Kampen J. J. A. van de Vijver D. A. M. C. Fraaij P. L. A. Haagmans B. L. Lamers M. M. Okba N. (2021). Duration and key determinants of infectious virus shedding in hospitalized patients with coronavirus disease-2019 (COVID-19). *Nat. Commun.* 12:267. 10.1038/s41467-020-20568-4 33431879 PMC7801729

[B169] Van Poelvoorde L. A. E. Saelens X. Thomas I. Roosens N. H. (2020). Next-generation sequencing: an eye-opener for the surveillance of antiviral resistance in influenza. *Trends Biotechnol.* 38 360–367. 10.1016/j.tibtech.2019.09.009 31810633

[B170] Vemula S. Zhao J. Liu J. Wang X. Biswas S. Hewlett I. (2016). Current approaches for diagnosis of influenza virus infections in humans. *Viruses* 8:96. 10.3390/v8040096 27077877 PMC4848591

[B171] Vijgen L. Keyaerts E. Moës E. Maes P. Duson G. Van Ranst M. (2005). Development of one-step, real-time, quantitative reverse transcriptase PCR assays for absolute quantitation of human coronaviruses OC43 and 229E. *J. Clin. Microbiol.* 43 5452–5456. 10.1128/JCM.43.11.5452-5456.2005 16272469 PMC1287813

[B172] Walper S. A. Lasarte Aragonés G. Sapsford K. E. Brown C. W. Rowland C. E. Breger J. C. (2018). Detecting biothreat agents: from current diagnostics to developing sensor technologies. *ACS Sensors* 3 1894–2024. 10.1021/acssensors.8b00420 30080029

[B173] Webster R. G. Govorkova E. A. (2014). Continuing challenges in influenza. *Ann. N. Y. Acad. Sci.* 1323 115–139. 10.1111/nyas.12462 24891213 PMC4159436

[B174] Welch N. L. Zhu M. Hua C. Weller J. Mirhashemi M. E. Nguyen T. G. (2022). Multiplexed CRISPR-based microfluidic platform for clinical testing of respiratory viruses and identification of SARS-CoV-2 variants. *Nat. Med.* 28 1083–1094. 10.1038/s41591-022-01734-1 35130561 PMC9117129

[B175] Wolters F. Grünberg M. Huber M. Kessler H. H. Prüller F. Saleh L. (2021). European multicenter evaluation of Xpert^®^ Xpress SARS-CoV-2/Flu/RSV test. *J. Med. Virol.* 93 5798–5804. 10.1002/jmv.27111 34050951 PMC8242864

[B176] Woo P. C. Y. Lau S. K. P. Lam C. S. F. Lai K. K. Y. Huang Y. Lee P. (2009). Comparative analysis of complete genome sequences of three avian coronaviruses reveals a novel group 3c coronavirus. *J. Virol.* 83 908–917. 10.1128/JVI.01977-08 18971277 PMC2612373

[B177] World Health Organization [WHO] (2020). *Laboratory Testing for Coronavirus Disease 2019 (COVID-19) in Suspected Human Cases.* 1–7. Geneva: World Health Organization.

[B178] World Health Organization [WHO] (2021). *WHO/2019-nCoV/Antigen_Detection/2021.1.* Geneva: World Health Organization

[B179] Wu C. Liu Y. Yang Y. Zhang P. Zhong W. Wang Y. (2020). Analysis of therapeutic targets for SARS-CoV-2 and discovery of potential drugs by computational methods. *Acta Pharm. Sin. B* 10 766–788. 10.1016/j.apsb.2020.02.008 32292689 PMC7102550

[B180] Wyllie A. L. Fournier J. Casanovas-Massana A. Campbell M. Tokuyama M. Vijayakumar P. (2020). Saliva or nasopharyngeal swab specimens for detection of SARS-CoV-2. *N. Engl. J. Med.* 383 1283–1286. 10.1056/NEJMc2016359 32857487 PMC7484747

[B181] Zambon M. Hays J. Webster A. Newman R. Keene O. (2001). Diagnosis of influenza in the community. *Arch. Intern. Med.* 161:2116. 10.1001/archinte.161.17.2116 11570941

[B182] Zhang F. Abudayyeh O. O. Gootenberg J. S. (2022). *A Protocol for Detection of COVID-19 Using CRISPR Diagnostics*. Available online at: https://www.broadinstitute.org/files/publications/special/COVID-19 detection (updated).pdf (accessed February 28, 2022).

[B183] Zhang W. Du R.-H. Li B. Zheng X.-S. Yang X.-L. Hu B. (2020). Molecular and serological investigation of 2019-nCoV infected patients: implication of multiple shedding routes. *Emerg. Microbes Infect.* 9 386–389. 10.1080/22221751.2020.1729071 32065057 PMC7048229

[B184] Zhang Y. Tanner N. A. (2021). Development of multiplexed reverse-transcription loop-mediated isothermal amplification for detection of SARS-CoV-2 and influenza viral RNA. *Biotechniques* 70 167–174. 10.2144/btn-2020-0157 33535813 PMC7860930

[B185] Zhao J. Liu J. Vemula S. V. Lin C. Tan J. Ragupathy V. (2016). Sensitive detection and simultaneous discrimination of influenza A and B viruses in nasopharyngeal swabs in a single assay using next-generation sequencing-based diagnostics. *PLoS One* 11:e0163175. 10.1371/journal.pone.0163175 27658193 PMC5033603

[B186] Zhen W. Manji R. Smith E. Wuitschick J. Lucic D. Berry G. J. (2022). Evaluation of the Alinity m Resp-4-Plex assay for the detection of severe acute respiratory syndrome coronavirus 2, Influenza A Virus, Influenza B virus, and respiratory syncytial virus. *Microbiol. Spectr.* 10:e0109021. 10.1128/spectrum.01090-21 35107357 PMC8809329

[B187] Zhou P. Yang X.-L. Wang X.-G. Hu B. Zhang L. Zhang W. (2020). A pneumonia outbreak associated with a new coronavirus of probable bat origin. *Nature* 579 270–273. 10.1038/s41586-020-2012-7 32015507 PMC7095418

[B188] Zhu N. Zhang D. Wang W. Li X. Yang B. Song J. (2020). A novel coronavirus from patients with pneumonia in China, 2019. *N. Engl. J. Med.* 382 727–733. 10.1056/NEJMoa2001017 31978945 PMC7092803

[B189] Ziegler K. Steininger P. Ziegler R. Steinmann J. Korn K. Ensser A. (2020). SARS-CoV-2 samples may escape detection because of a single point mutation in the N gene. *Euro Surveill.* 25:2001650. 10.2807/1560-7917.ES.2020.25.39.2001650 33006300 PMC7531073

